# Samarium-Driven Vacancy Engineering in CeO_2_/Ru(0001) Inverse Catalysts

**DOI:** 10.1021/acs.chemmater.6c00161

**Published:** 2026-07-29

**Authors:** Raquel Sánchez-Barquilla, Alexander Contreras-Payares, Rudi Tschammer, Carlos Morales, Lars Buß, Björn Riedel, Iulia Cojocariu, Tevfik Onur Menteş, Matteo Jugovac, Pablo G. Lustemberg, Andrea Locatelli, M. Verónica Ganduglia-Pirovano, Jan Ingo Flege

**Affiliations:** † Applied Physics and Semiconductor Spectroscopy, 38871 Brandenburg University of Technology Cottbus-Senftenberg, Cottbus 03046, Germany; ‡ 83076 Instituto de Catálisis y Petroleoquímica, (ICP-CSIC), C/Marie Curie 2, Madrid 28049, Spain; § Ph.D. Theoretical Chemistry and Computational Modelling Doctoral School, Universidad Autónoma de Madrid, C/Francisco Tomás y Valiente 2, Ciudad Universitaria de Cantoblanco, Madrid 28049, Spain; ∥ 18474 Elettra-Sincrotrone Trieste, S.C.p.A, Strada Statale 14 - km 163.5 in AREA Science Park, Basovizza, Trieste 34149, Italy

## Abstract

Ceria-based inverse catalysts derive their functionality from Ce^3+^–oxygen vacancy chemistry, yet strategies to deliberately control vacancy formation and distribution at metal–oxide interfaces remain limited. Here, we demonstrate that aliovalent samarium doping provides a direct and tunable route to vacancy engineering in the prototypical CeO_2_/Ru­(0001) inverse catalyst. Using real-time, nanometer-resolved low-energy electron microscopy in combination with low-energy electron microdiffraction, X-ray absorption spectroscopy in photoemission electron microscopy, and density functional theory, we directly correlate lattice structure, oxidation state, and vacancy formation under reducing conditions. Sm incorporation generates *extrinsic* oxygen vacancies, which are accompanied by Ce^3+^ formation, which induces a pronounced lattice expansion that in turn fosters the formation of additional *intrinsic* oxygen vacancies, substantially enhancing reducibility and stabilizing reduced ceria phases relative to undoped CeO_2_. The doped system exhibits accelerated reduction kinetics, ordered intrinsic vacancy formation, and modified phase-transition behavior. By establishing how aliovalent dopants govern the vacancy landscape and redox response in inverse ceria catalysts, this work provides atomic-level mechanistic insight and concrete design principles for engineering reducible oxides at metal–oxide interfaces with tailored redox properties.

## Graphical abstract

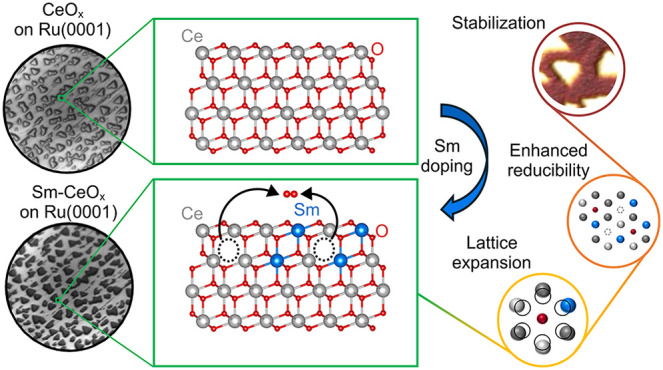

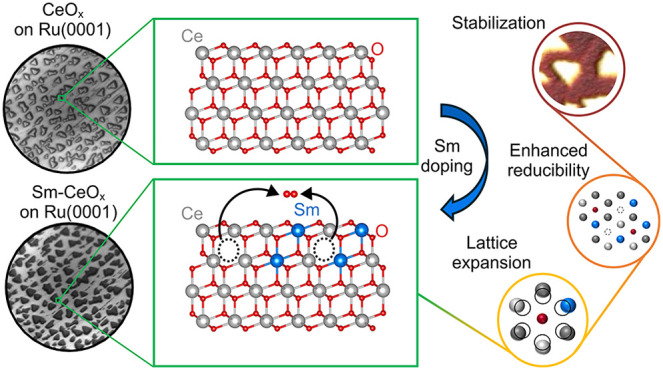

## Introduction

Ceria is a benchmark redox-active oxide whose ability to cycle between Ce^4+^ and Ce^3+^ ^1–3^ underpins its performance in catalytic transformations^4–6^ such as CO oxidation^7,8^ the water–gas-shift reaction,^7,9,10^ and CO_2_ conversion.^11–13^ Its fluorite lattice accommodates oxygen vacancies^14^ with relatively low energetic cost^15–18^ making defect formation, migration, and reoxidation central to its catalytic behavior. As a result, controlling the oxygen-vacancy landscape in ceria has become a powerful means for tuning activity and selectivity across a range of reactions.

Inverse catalysts^19–24^ based on ceria nanostructures supported on metals^25^ provide a model platform for probing vacancy–metal interactions with nanoscale spatial and chemical resolution.^26–35^ In the prototypical CeO_2_/Ru­(0001) system^27,36–41^ CeO_2_(111) nanoislands undergo well-defined reduction pathways that produce ordered vacancy superstructures and phase transformations to cubic and hexagonal Ce_2_O_3_ polymorphs.^29,31,42^ These behaviors provide an ideal testbed for understanding how dopants reshape the vacancy energy landscape under reactive environments.

Aliovalent doping^43^ is an established strategy for stabilizing oxygen vacancies in bulk and nanoparticulate ceria.^44–49^ Rare-earth dopants, such as Sm^3+^, substitute for Ce^4+^, generating *extrinsic* oxygen vacancies, with charge compensation accommodated by Ce^3+^ or Sm^3+^. Here, we adopt the standard defect-chemistry nomenclature, where extrinsic oxygen vacancies are those formed to compensate the charge imbalance introduced by aliovalent substitution, while *intrinsic* oxygen vacancies correspond to native defects of the ceria lattice formed independently of dopant charge compensation.^50,51^ The associated formation of Ce^3+^ induces a lattice expansion, which is expected to lower the formation energy of *intrinsic* vacancies. From a theoretical perspective, first-principles studies have shown that aliovalent doping modifies both the lattice structure and the stability of oxygen vacancies in CeO_2_, with La-, Gd-, and Sm-doped systems serving as key model cases.^52–54^ While these effects are well documented for powders and bulk samples^55,56^ their manifestation in well-defined inverse catalysts^57–59^ —where lattice strain, reduced dimensionality, and interfacial coupling are more pronounced and experimentally accessible—remains insufficiently understood.

Low-energy electron microscopy (LEEM) and microdiffraction (μLEED)^60^ allow real-time monitoring of the structural evolution of oxide films and nanostructures^61^ during redox cycling, both in real-space with nanometer resolution and in reciprocal space at the atomic scale. When combined with X-ray absorption spectroscopy in photoemission electron microscopy (XAS-PEEM), which provides element-specific oxidation-state mapping, these techniques yield a uniquely comprehensive view of structure–vacancy–redox coupling in catalytically relevant environments. Yet, such multimodal correlative measurements have rarely been applied to doped ceria interfaces.

Here, we use LEEM, μLEED, XAS-PEEM, X-ray photoemission electron microscopy (XPEEM), and density functional theory (DFT) to elucidate how samarium doping restructures the vacancy energy landscape and redox behavior of CeO_2_/Ru­(0001). We show that postannealing ceria with metallic Sm leads to substantial dopant incorporation, lattice expansion associated with Ce^3+^ formation, and the creation of extrinsic vacancies, while simultaneously lowering the formation energy of intrinsic vacancies under reducing conditions. Real-time *I­(V)*-LEEM measurements^62–64^ reveal accelerated reduction kinetics and altered phase-transition pathways compared with undoped CeO_2_, whereas DFT connects these behaviors to charge-compensation energetics and dopant-induced strain. Together, these results establish doped inverse catalysts as powerful model systems for resolving how aliovalent dopants regulate vacancy chemistry at oxide–metal interfaces and for improving redox activity by rational catalyst design.

## Results and Discussion

In the first part of this section, we examine the preparation of Sm-doped ceria, characterizing its morphology and structure using low-energy electron microscopy (LEEM), microspot low-energy electron diffraction (μLEED), and *I­(V)*-LEEM analysis. The oxidation state of the metal cations within the oxide islands is then probed by X-ray absorption spectroscopy in photoemission electron microscopy (XAS-PEEM), and the experimental results are correlated with density functional theory (DFT) calculations.

In the second part, we address the reduction behavior of Sm–CeO_*x*_ nanoislands when exposed to hydrogen at a partial pressure of 5 × 10^–6^ mbar and subsequently annealed to 700 °C under ultrahigh vacuum (UHV) conditions. The reduced doped structures are compared with undoped CeO_2_/Ru­(0001) subjected to analogous treatments.^29,31^

### Structural Characterization of As-Grown Particles

The CeO_2_/Ru­(0001) system was prepared following established procedures.^38^ Briefly, cerium was evaporated onto the clean Ru(0001) substrate heated to 800 °C in 5 × 10^–7^ mbar O_2_ to ensure complete oxidation of the deposited metal (cf. Supporting Information, Sec. One and Figures S1 and S2). Under these conditions, CeO_2_ forms triangular (111)-oriented islands several hundred nanometers in size, appearing in two types of rotational domains (Figure 1(a)), whose shapes depend on the step direction and step height of the crystalline support.^39^

**Figure 1:**
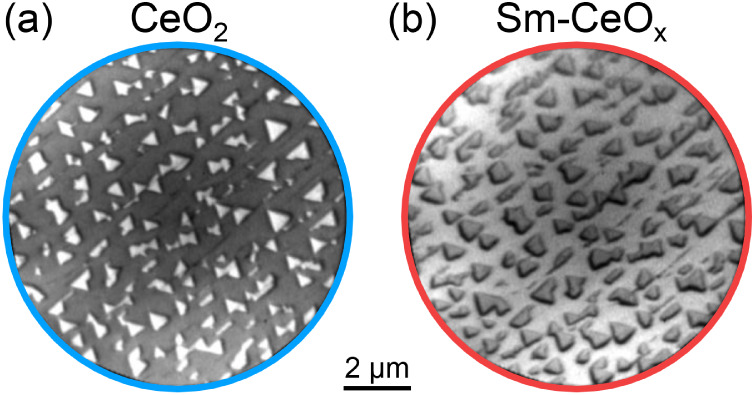
LEEM images at 8.2 eV of as-grown nanoislands on Ru(0001) measured at 800 °C. (a) CeO_2_ nanoislands exhibit their characteristic triangular shape. (b) Postannealing of the CeO_2_ particles in (a) with metallic Sm results in a darker appearance of the particles at this energy.

Metallic Sm was then deposited onto the CeO_2_ islands by physical-vapor deposition at 800 °C sample temperature under UHV conditions. The change in contrast of the ceria islands in the LEEM images (Figure 1(b)) signals the presence of Sm and indicates the formation of Sm-CeO_*x*_ islands. Spatially resolved XAS-PEEM measurements at the Sm M_5_ edge confirm that Sm resides predominantly on the ceria islands (cf. Supporting Information, Sec. Two and Figure S3). A 7% Sm concentration within the nanoislands is estimated from a spatially resolved, quantitative analysis of the Sm 4d and Ce 4d regions extracted from X-ray photoemission electron microscopy (XPEEM) data (cf. Supporting Information, Sec. Three and Figure S4).

Figure 2 displays μLEED patterns from the areas shown in Figure 1(a) and (b). Before Sm deposition, the ceria islands show the characteristic (1.40 × 1.40) superstructure of the (111)-oriented fluorite lattice (blue line in Figure 2). After Sm deposition, the diffraction pattern remains hexagonal and aligned with Ru(0001), but the in-plane lattice parameter increases to 3.97 ± 0.07 Å (red line) relative to CeO_2_(111) (3.82 Å).[Fn fn1] Because Sm_2_O_3_ grown under identical conditions has a smaller in-plane lattice parameter (3.70 ± 0.10 Å, cf. Supporting Information, Sec. Four and Figure S5), in agreement with previously reported values for Sm_2_O_3_/Ru­(0001) (3.74 Å, see ref 65) and Sm_2_O_3_/Pt­(111) (3.82 Å, see ref 66), the expansion observed here strongly indicates that Sm is incorporated into the ceria lattice rather than forming an Sm_2_O_3_ overlayer.

**Figure 2:**
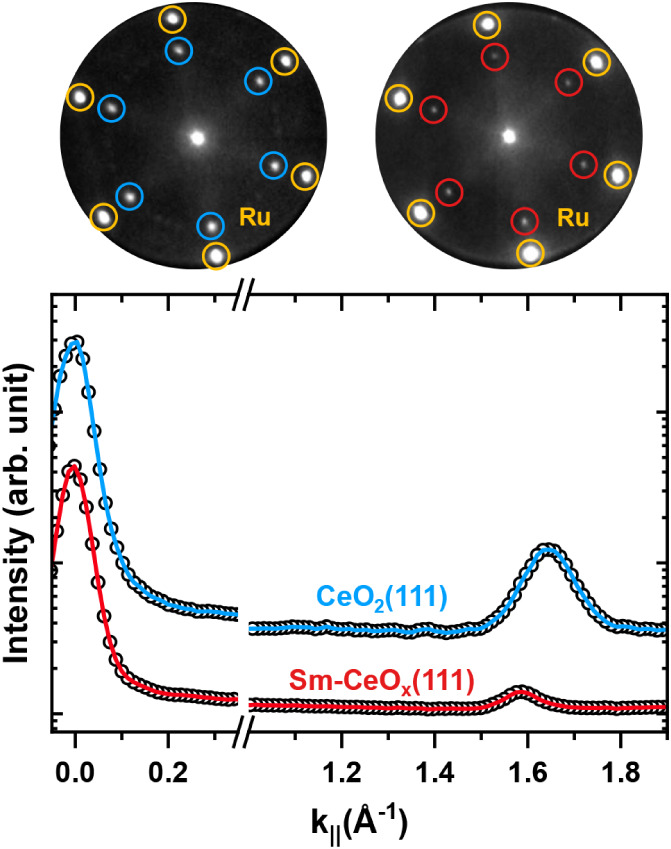
Top: μLEED patterns acquired from CeO_2_ nanoislands (blue), and the same CeO_2_ nanoislands after postannealing with Sm (red). Ru(0001) substrate reflections are marked in yellow. Bottom: Corresponding LEED intensity profiles, vertically offset for clarity.

The magnitude of the lattice expansion in aliovalent rare-earth doping of fluorite structures has been related to both the dopant ionic radius^67–70^ and the formation of associated extrinsic oxygen vacancies.^67^ Here, however, the expansion is primarily attributed to Ce^3+^ formation associated with oxygen vacancies. In our case, the measured expansion of 3.8% after Sm deposition is consistent with the ∼3–4% lattice expansion associated with the fluorite-to-bixbyite transition in fully reduced ceria (Ce_2_O_3_),^15^ indicating a high degree of reduction of the ceria nanoislands.

Further insight into particle structure and oxidation state comes from *I­(V)*-LEEM. The *I­(V)* curve shape reflects the local electron reflectivity as a function of the kinetic energy^63,71^ providing a sensitive fingerprint of composition, crystallographic orientation, and oxidation state. Figure 3 shows the *I­(V)* curves measured for the Sm-CeO_*x*_ nanoislands together with those of CeO_2_(111) and Sm_2_O_3_(111) reference systems (cf. Supporting Information, Sec. 4), as well as previously measured reference curves^29^ and calculated ones obtained from *ab initio* scattering calculations.^65^

**Figure 3:**
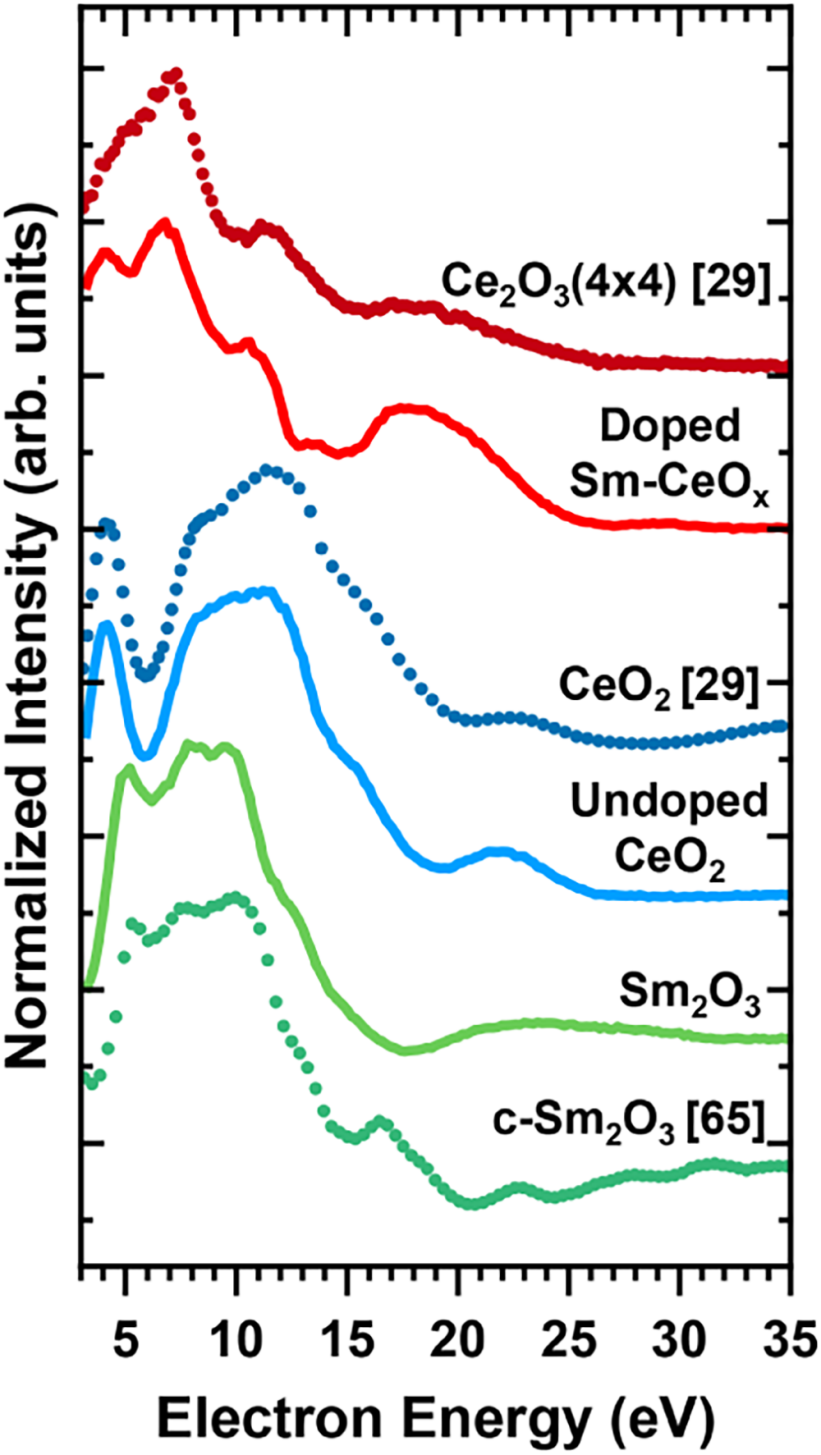
I­(V) curves acquired on top of the different nanoislands (solid lines), compared with calculated and measured reference curves (dotted lines). Curves are vertically offset for clarity. Reference Ce_2_O_3_ and CeO_2_ adapted with permission from ref 29. Copyright [2015] [JOHN Wiley & SONS]. Reference c-Sm_2_O_3_ adapted from ref 65. Copyright [2023] [ELSEVIER BV]).

Importantly, the *I­(V)* curve of the ceria islands changes markedly after Sm postdeposition at 800 °C. Control measurements of undoped CeO_2_ nanoislands under UHV conditions show that the change in the *I­(V)* curve is not a temperature effect (see Supporting Information, Sec. Five and Figure S6). The initially fully oxidized CeO_2_ nanoislands (solid blue line in Figure 3) acquire an *I­(V)* shape resembling that of fully reduced Ce_2_O_3_ ^29^ (red solid line), rather than exhibiting the spectral features characteristic of cubic Sm_2_O_3_ (green solid line). Independent Sm_2_O_3_(111) reference particles grown under identical conditions (see Supporting Information, Sec. Four and Figure S5) confirm the expected samaria fingerprint. The absence of these features in the Sm-CeO_*x*_ islands indicates that Sm does not remain as a surface oxide but instead becomes incorporated into the ceria lattice, in agreement with the μLEED analysis.

Although the evaporation of metallic Sm under UHV conditions and its subsequent oxidation to Sm^3+^ in the Sm-CeO_*x*_ islands resembles the mechanism reported for the Ce-ceria interaction, where metallic Ce deposited on ceria extracts oxygen from the surface layer, thereby inducing reduction^42,72^ the behavior observed here is markedly different. The absence of Sm_2_O_3_-like fingerprints in the *I­(V)* curves, together with the lack of contrast inhomogeneities in the LEEM images (Figure 1), rules out the formation of Sm_2_O_3_ at the surface. Instead, Sm cations diffuse into and substitute within the ceria lattice, thereby preserving the fluorite structure.

Doping with aliovalent cations such as Sm^3+^ is known to introduce extrinsic oxygen vacancies and to facilitate the formation of additional (intrinsic) vacancies under reducing conditions.^44,45,48,70,73–75^ The *I­(V)* curve of the reduced Sm–CeO_*x*_ islands therefore likely reflects a combination of extrinsic vacancies created by Sm incorporation and intrinsic vacancies formed during the high-temperature preparation. The absence of vacancy-ordering superstructure spots in the μLEED pattern of the as-prepared Sm-CeO_*x*_ islands (Figure 2) shows that these vacancies remain disordered.

The reduction effect is further corroborated by XAS-PEEM measurements (Figure 4). Analyzing the Ce M_5_ absorption spectra after the Sm deposition confirms the presence of a moderate amount of Ce^3+^ (Figure 4(a)). Fitting the data using a linear combination of Ce^3+^ (dark blue) and Ce^4+^ (light blue) reference spectra,^29,76,77^ we estimate the average amount of Ce^3+^ in the islands to about 18 ± 2%. The Sm measured at the Sm M_5_ edge is detected exclusively as Sm3+^65^ (Figure 4(a), inset). Note that, in a simplified interpretation, the comparison between the Ce^3+^ concentration determined from XAS and the ∼7% Sm concentration obtained from XPEEM might suggest that oxygen vacancies are formed in association with Sm^3+^ incorporation. However, the measured Ce^3+^ fraction exceeds the Sm concentration, indicating that extrinsic vacancies alone cannot account for the observed reduction. This implies that, in addition to dopant-induced (extrinsic) vacancies, a significant number of intrinsic oxygen vacancies must be present, most likely due to the high temperature applied during Sm deposition. In addition, the Ce^3+^ concentrations derived from XAS and the more surface-sensitive *I­(V)*-LEEM are not directly comparable due to their different probing depths, with *I­(V)*-LEEM indicating a higher degree of reduction near the surface.

**Figure 4:**
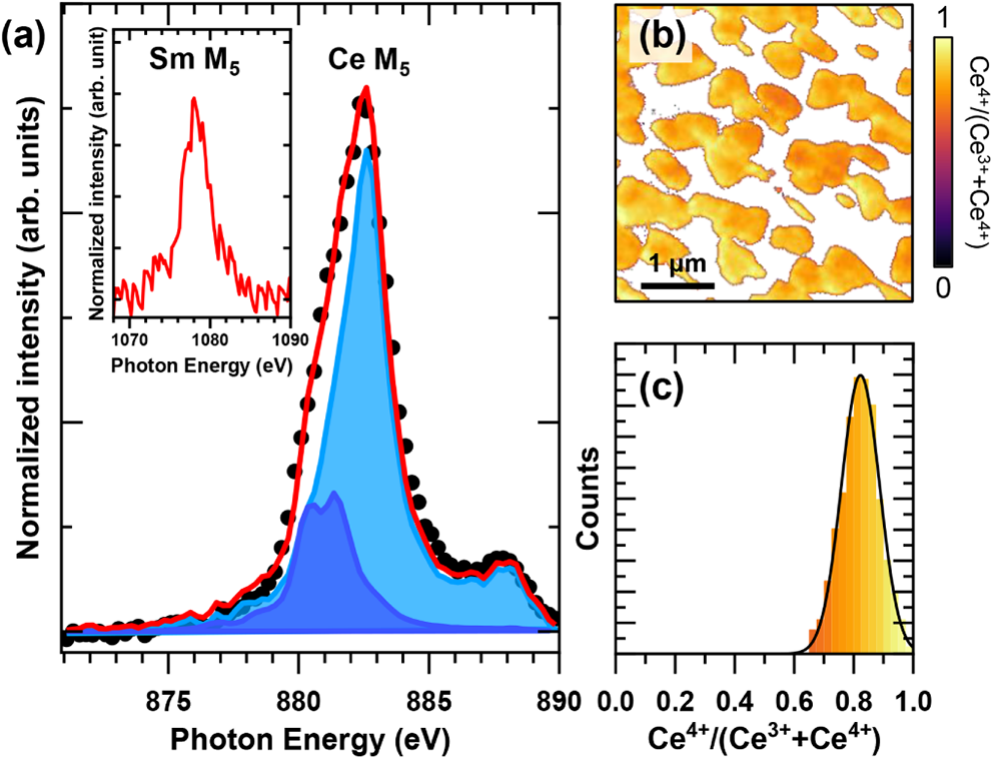
(a) X-ray absorption spectra at the Ce M_5_ edge, extracted from the Sm-CeO_x_ nanoislands. The acquired spectrum (black dots) is fitted (red line) by a linear combination of Ce^3+^ (dark blue) and Ce^4+^ (light blue) component reference spectra, yielding a Ce^4+^ atomic concentration of (82 ± 2)%. In the inset, an X-ray absorption spectrum recorded at the Sm M_5_ edge and obtained from the same nanoislands is displayed. (b) False-color representation of the results from linear combination fitting the XAS spectra at each pixel with the same reference spectra as shown in (a). Lighter colors indicate a higher amount of Ce^4+^. (c) Histogram of the relative amount of Ce^4+^ taken over the area shown in (b). The distribution is fitted to a Gaussian function (black line). This histogram gives an overall amount of Ce^4+^ of 0.82 ± 0.06.

The spatial distribution of the cerium oxidation state is mapped by extracting and fitting the ceria XAS data at every image pixel of our measurement, the result of which is plotted in false color in Figure 4(b). Obviously, reduction is essentially homogeneous, as the spatial variation in oxidation state is rather narrow (see histogram in Figure 4(c)) and takes place across the entire surface of the nanoislands. Because the probing depth of XAS-PEEM is more than one order of magnitude larger than that of the electrons used in *I­(V)*-LEEM, the XAS spectra represent an average oxidation state across the entire island thickness and therefore indicate that the nanoislands are only mildly reduced overall.

Together, the *I­(V)*-LEEM, μLEED, XPEEM, and XAS-PEEM measurements show that Sm incorporation reshapes the vacancy formation and redox behavior of the ceria islands, resulting in facile vacancy creation, partial Ce reduction, and lattice expansion associated with Ce^3+^ formation. These effects highlight the strong coupling between doping, local structure, and reducibility that is most prominent at the catalyst surface, extending observations for aliovalent bulk doping in fluorite oxides. In the following, we turn to DFT calculations to unravel the underlying atomic-scale mechanisms, which likely involve a combination of charge compensation and strain-assisted vacancy formation, and to gain deeper insights into the energetics and structural consequences of Sm doping, substantiating the mechanistic picture suggested by the experiments.

### Oxygen Vacancy Formation and Energetics in Sm-CeO*_x_*

To rationalize the experimental observation that Sm incorporation reshapes the vacancy formation and redox behavior of ceria, we carried out DFT calculations to examine oxygen vacancy formation in both undoped and Sm-doped CeO_2_(111) surfaces. In stoichiometric ceria, removal of a neutral oxygen atom leaves two excess electrons that localize on Ce^4+^ cations, reducing them to Ce^3+^.^16,78–80^ These electrons preferentially occupy next-nearest-neighbor (NNN) Ce sites relative to the vacancy, and the most stable configuration corresponds to a subsurface oxygen vacancy. For example, formation of an intrinsic subsurface vacancy in CeO_2_(111) with (2 × 2) periodicity, $V_{\mathrm{SS}}^{[2,2̲]}$ , with both Ce^3+^ ions located in NNN cation sites, one in the second and the other in the fifth atomic layer, [2, 2], requires 1.98 eV (Figures 5, S7­(a), and S8), in line with previous works.^16,78–80^ This value provides a reference for the intrinsic vacancy formation energy in undoped ceria.

**Figure 5:**
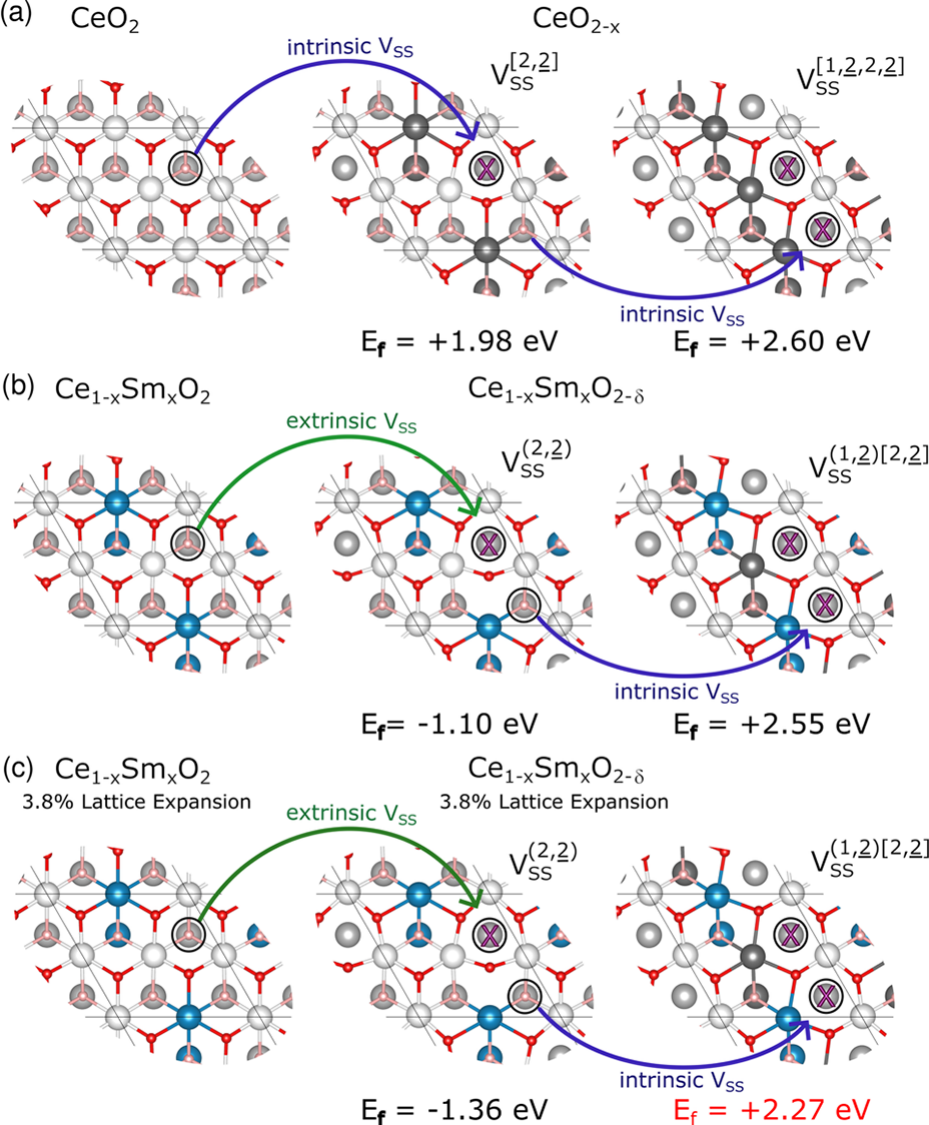
DFT-calculated formation energies, *E_f_*, for subsurface oxygen vacancies in (a) CeO_2_(111), (b) Sm-doped Ce_1–*x*_Sm*_x_* O_2_(111), and (c) Sm-doped Cee_1–*x*_Sm*_x_* O_2_(111) with a 3.8% lattice expansion. Color code: surface O atoms in red, subsurface O atoms in light red, Ce^4+^ in white (light gray for Ce^4+^ in lower CeO_2_ trilayers), Ce^3+^ in dark gray, and Sm dopants in blue. For clarity, only atoms in the first O–Ce–O trilayer (TL) and cations in the second TL are shown. The optimized structures were visualized and the images generated using the VESTA software.^86^

When a Sm^3+^ ion substitutes for Ce^4+^ in the cation sublattice, the situation changes markedly. Formation of an extrinsic subsurface oxygen vacancy becomes thermodynamically favorable. In the most stable configuration, $V_{\mathrm{SS}}^{(2)[2̲]}$ , the calculated formation energy is E_f_ = −1.15 eV ((Figure S7b)), indicating that this charge-compensating (extrinsic) vacancy is thermodynamically favorable. In this case, only one Ce^3+^ cation is generated, because the aliovalent Sm^3+^ partially compensates the excess charge associated with oxygen removal. Comparable DFT + U studies on Sm-doped ceria report vacancy formation energies that depend sensitively on the degree of charge compensation and on the local dopant–vacancy geometry (Table S1 in the Supporting Information). For bulk-like Sm–CeO_2_, Zhang et al.^81^ reported a mildly exothermic value (E_f_ ≈ −0.30 eV) for a partially compensated extrinsic vacancy, without explicitly resolving the accompanying Ce^3+^ configuration. For Sm–CeO_*x*_(111), Liu et al.^82^ obtained E_f_ ≈ 1.68 eV for a surface oxygen vacancy adjacent to Sm, a value that contrasts with the expected stabilization of charge-compensated extrinsic vacancies, underscoring the sensitivity of these energies to the local vacancy configuration and to details of the computational treatment. The substantially lower formation energy obtained here (E_f_ = −1.15 eV) for a subsurface vacancy is consistent with a concerted stabilization arising from (i) the intrinsic preference for subsurface vacancy formation and (ii) the placement of both the Sm^3+^ dopant and the reduced Ce^3+^ ion at next-nearest-neighbor positions relative to the vacancy, closely mirroring the most stable intrinsic-vacancy arrangement in undoped CeO_2_(111).

We next examined configurations containing two Sm^3+^ dopants and one extrinsic subsurface vacancy (Figure S9). The lowest energy arrangement, $V_{\mathrm{SS}}^{\left(2,2̲\right)}$ , places both Sm cations in NNN positions relative to the vacancy, one in the second and the other in the fifth atomic layer, (2, 2). In this configuration, no Ce^3+^ is formed because the two Sm^3+^ cations fully compensate the excess charge. Structurally, the relaxed Sm-doped configuration closely resembles the intrinsic subsurface vacancy structure in undoped CeO_2_ $\left(V_{\mathrm{SS}}^{[2,2̲]}\right)$ , as shown in Figure 5(b). This behavior is consistent with prior DFT + U studies showing that trivalent Sm doping can stabilize fully charge-compensated extrinsic oxygen vacancies in bulk Sm-CeO_2_, yielding negative vacancy formation energies (see Table S1).^52,54,83^ A detailed discussion of the configurations examined and their relative energies is provided in the (Supporting Information Sec. 6, Figure S9 and S10).

To assess how doping affects the formation of intrinsic vacancies, we calculated the formation energies of a second oxygen vacancy in the doped system. As in undoped CeO_2_,^78^ this vacancy also prefers a subsurface site. The lowest-energy configuration for this second vacancy is $V_{\mathrm{SS}}^{(1,2̲)[2,2̲]}$ , with a formation energy of 2.55 eV. In this arrangement, both Ce^3+^ ions occupy NNN cation sites, one in the second and the other in the fifth atomic layer ([2, 2]; see Figure 5(b) and S11). The Sm^3+^ dopants are located in a nearest- and a next-nearest-neighbor cation site, specifically in the second and fifth atomic layers, respectively, (1, 2). The formation energy is only slightly lower than that of the corresponding intrinsic vacancy in undoped CeO_2_, $V_{\mathrm{SS}}^{[1,2,2̲,2̲]}$ , which has a value of 2.60 eV (Figures 5(a) and 5(b)). A reduction in the overall vacancy concentration yields the same qualitative trend (Figure S8). This result is consistent with previous DFT + U calculations of intrinsic vacancy formation in bulk Sm–CeO_2_, which yield comparable energies. Lucid et al.^84^ reported E_f_ = 2.76 eV for a bulk configuration with two Sm dopants in NN and NNN cation sites relative to the vacancy and Ce^3+^ localized at NN sites, while An et al.^54^ obtained E_f_ = 2.85 eV for a related bulk arrangement with Sm dopants at NNN positions and Ce^3+^ also adjacent to the vacancy (Table S1). Notably, most other theoretical works focus on the first, charge-compensated (extrinsic) vacancy and do not explicitly report the energetic cost of forming an additional (intrinsic) vacancy.

μLEED experiments reveal a 3.8% lattice expansion upon Sm incorporation. When this expansion is included in the DFT model, the formation energy of the same intrinsic vacancy drops substantially to 2.27 eV (Figure 5(c)). This demonstrates that lattice strain plays a key role in enhancing reducibility under reducing conditions. This trend is consistent with a previous report showing that tensile strain reduces the formation energy of oxygen vacancies in CeO_2_(111) surfaces.^85^

In that study, a 4% increase in lattice parameter decreased the vacancy formation energy by 37.5%, reflecting the same qualitative behavior as the 12.7% reduction observed here for Sm-induced strain. Similarly, Grieshammer et al.^53^ demonstrated in bulk CeO_2_ that a 3–4% expansion can lower E_f_ by up to 35%, providing a direct theoretical basis for the strain-assisted vacancy formation observed in our system.

Mechanistically, the expanded lattice partially alleviates the strain introduced by Ce^3+^ formation and, to a lesser extent, by the presence of Sm^3+^, thereby lowering the energetic cost of vacancy formation. In the relaxed configuration, both Sm^3+^ and Ce^3+^ ions occupy next-nearest-neighbor (NNN) positions relative to the vacancy, further minimizing local distortions and electrostatic repulsion, in line with the defect-pairing geometry predicted by Grieshammer et al.^53^

The replacement of Ce^4+^ by Sm^3+^ ions affects reducibility through two coupled contributions. First, local charge compensation makes the formation of the extrinsic oxygen vacancy thermodynamically favorable. Second, the associated formation of Ce^3+^ induces lattice expansion, which significantly lowers the formation energy of an additional intrinsic vacancy by reducing the associated strain energy. Thus, while Sm substitution directly promotes extrinsic vacancy formation, the enhanced reducibility associated with intrinsic vacancy formation is mainly governed by the resulting lattice expansion. This establishes a self-reinforcing mechanism linking Sm doping, Ce^3+^ formation, lattice expansion, and enhanced intrinsic vacancy formation. Additional calculations disentangling the effects of lattice expansion and dopant concentration are provided in the (Supporting Information Figure S12). As such, the Sm–CeO_*x*_ system represents a particularly tunable platform for oxygen-vacancy engineering, with implications not only for fundamental oxide chemistry but also for catalytic processes where vacancy-mediated mechanisms dominate.

In the following, we expose the Sm-CeO_*x*_/Ru­(0001) system at moderate temperature to molecular hydrogen and elevated temperature under UHV conditions, and experimentally assess its reducibility at the nanometer scale using *in situ* dynamic *I­(V)*-LEEM, directly comparing the results with observations from the undoped CeO_*x*_/Ru­(0001) system.

### Reduction of Sm-CeO*_x_* Nanoislands

The formation of intrinsic oxygen vacancies under reducing conditions was investigated by exposing Sm-CeO_*x*_ islands to hydrogen at 5 × 10^–6^ mbar for 30 min, under conditions comparable to those used previously for undoped CeO_2_/Ru­(0001).^29^ To prevent surface contamination, the sample was maintained at 400 °C before and during H_2_ exposure. Reduction was monitored in real-time using dynamic *I­(V)*-LEEM curves, with the specular electron reflectivity continuously recorded between 0 and 20 eV (Figure 6(a)). This approach enables tracking of structural^62,64^ and oxidation-state changes^27,29,31^ from modifications in the *I­(V)* spectral line shape during hydrogen exposure.

**Figure 6:**
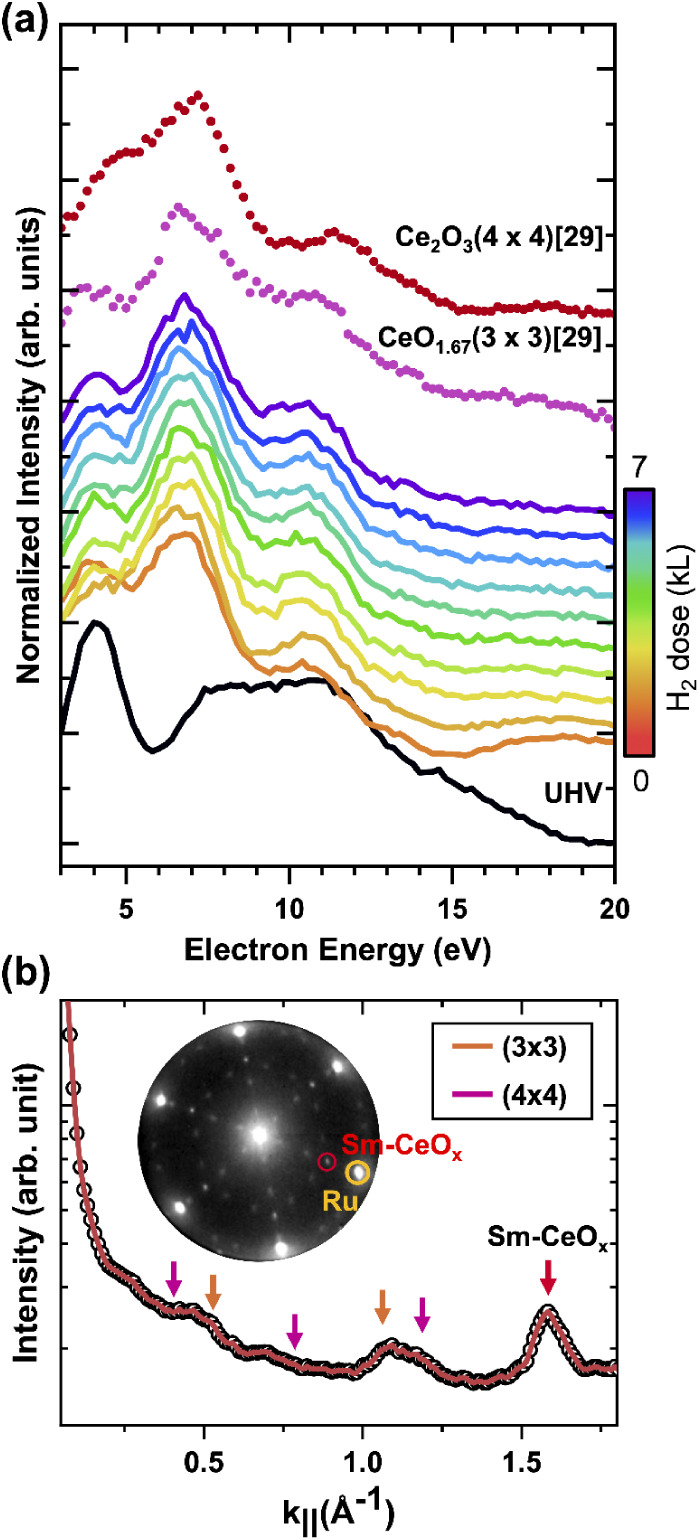
(a) Dynamic I­(V)-LEEM curves of Sm-CeO*_x_* nanoislands recorded before (black) and during H_2_ exposure. The colorbar indicates cumulative H_2_ dose. Dotted lines show reference curves. (b) Radial profile extracted from the μLEED pattern (shown in the inset) acquired during H_2_ reduction, with Sm-CeO*_x_* and substrate integer reflections marked by red and yellow circles, respectively. The positions of the superstructure spots corresponding to the (3 × 3) and (4 × 4) reconstructions are indicated by orange and magenta arrows in the profile, respectively. Reference Ce_2_O_3_ (4 × 4) and CeO_1.67_ (3 × 3) adapted with permission from ref 29. Copyright [2015] [JOHN Wiley & SONS].

Immediately before H_2_ introduction, the Sm-CeO_*x*_ islands exhibited an *I­(V)* signature (black curve in Figure 6(a)) similar to that of CeO_1.8_,^31^ consistent with mild reoxidation upon cooling to 400 °C in UHV after deposition of metallic Sm at 800 °C. At this stage, the vacancy population is dominated by extrinsic vacancies associated with Sm doping, although some intrinsic vacancies may already be present due to the high-temperature preparation. Upon H_2_ exposure, the nanoislands reduced almost instantaneously. At a dose of 2 kL (yellow curve), the *I­(V)* profile matched that of partially reduced ceria with CeO_1.67_,^29^ after which no further evolution was observed, indicating a stable oxidation state. Notably, the *I­(V)* signature of fully reduced Ce_2_O_3_ was never reached. Furthermore, the Sm–CeO_*x*_ islands reduce uniformly across their entire area, consistent with Sm incorporation within the ceria lattice and in contrast to undoped CeO_2_/Ru­(0001), which exhibits pronounced spatial reduction inhomogeneities under similar conditions.^29^

Chemical reduction was further corroborated by μLEED. After H_2_ exposure, the diffraction pattern (Figure 6(b), inset) displayed superstructure spots characteristic of ordered oxygen vacancies. A radial profile taken in the direction of these spots (Figure 6(b)) shows that the superstructure spots lie between the positions expected for the (3 × 3) and (4 × 4) phases that form during reduction of undoped CeO_2_ ^29,42^ which are indicated by the orange and magenta arrows, respectively. Such an interplay of phases has already been observed for CeO_2_/Ru­(0001): Moderate H_2_ doses and pressures yield a commensurate (3 × 3) superstructure;^29^ upon increasing pressure, the (3 × 3) spots move apart in reciprocal space, and additional spots emerge at the half-integer position between them. This evolution culminates in the (4 × 4) superstructure associated with fully reduced, cubic Ce_2_O_3_(111). Here, with Sm incorporation, we observe a snapshot compatible with a similar sequence of superstructures, showcasing that the reduction–oxidation dynamics are markedly accelerated, while the underlying reduction pathway is largely preserved.

The significantly enhanced reducibility of Sm-CeO_*x*_ becomes obvious in isothermal reduction experiments at 700 °C under UHV conditions, where reduction is almost instantaneous and spatially inhomogeneous: Figure 7(a) shows a LEEM image acquired at 8.2 eV during the first minute of thermal reduction. Correspondingly, contrast variations occur across the islands, reflecting different reduction levels within the islands, with certain regions reducing substantially faster than others. Unlike in the undoped system, where heating to 700 °C leads to a gradual, homogeneous transformation from CeO_2_(111) to c-Ce_2_O_3_(111) islands over roughly 80 min,^31^ no extended c-Ce_2_O_3_(111) domains were detected here. Instead, *I­(V)* spectra collected at different positions show clear similarities with fully reduced a-Ce_2_O_3_(0001) areas (orange line, Figure 7(b)), coexisting with partially reduced c-CeO_*x*_(111) regions of varying reduction levels (yellow and blue lines).[Fn fn2] Yet, the nanoislands do not shrink visibly, much unlike in the reference experiment.^31^ Without Sm, upon annealing for more than 80 min, a phase transition from CeO_2_(111) to coexisting, extended c-Ce_2_O_3_(111) and a-Ce_2_O_3_(0001) domains occurs, accompanied by partial decomposition of cerium oxide into volatile oxygen and metallic Ce, which subsequently spills onto the Ru surface and nucleates into a disordered Ce adlayer. Hence, Sm incorporation suppresses both partial dissolution and Ce redistribution.

**Figure 7:**
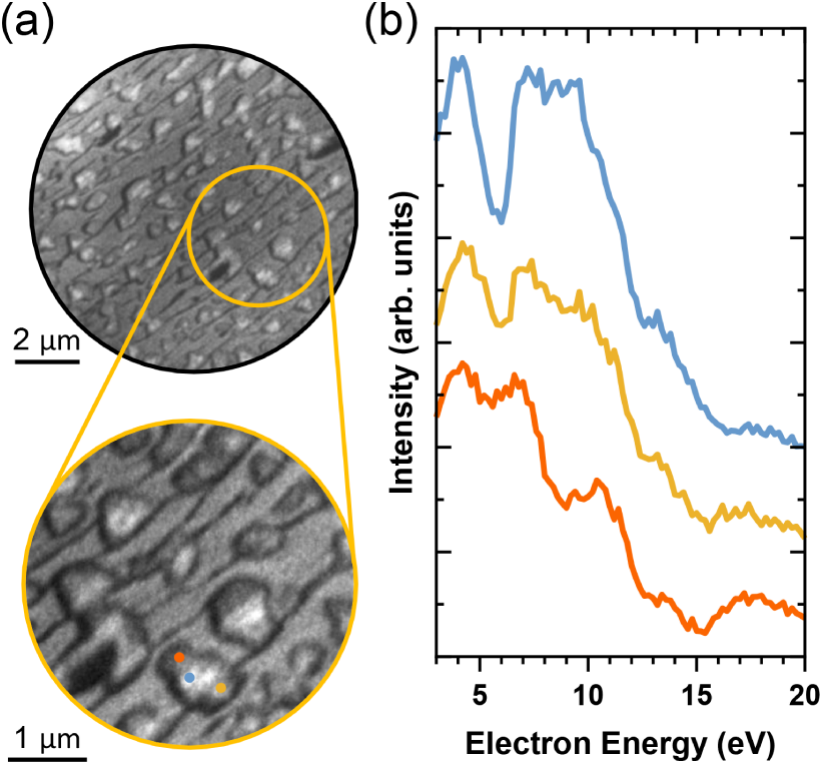
(a) LEEM image acquired at 8.2 eV during annealing at 700 °C of Sm-CeO*_x_* nanoislands. Variations in brightness within the individual islands reflect regions reducing at different rates. Colored circles mark the locations from which the *I­(V)* curves in panel (b) were obtained. (b) *I­(V)* curves taken from distinct regions of a single Sm-CeO*_x_* island. Spectra are vertically shifted for clarity.

A third, related effect of Sm doping is evident in the *I­(V)* curves extracted before annealing (solid red line), during steady-state reduction (solid orange), and after cooling to 400 °C (solid magenta), as shown in Figure 8. Even though the changes in the *I­(V)* line shape are subtle, they unambiguously signal a phase transition of the nanoislands from cubic CeO_2_(111) to the a-Ce_2_O_3_(0001) structure with type-(I) termination at 700 °C, consistent with the reference spectra^31^ (dotted orange lines). Upon cooling back to 400 °C under UHV conditions, the *I­(V)* profile reverts to that characteristic of cubic CeO_*x*_(111), with *x* ∼ 1.75.^31^ These observations are consistent with enhanced vacancy mobility, which facilitates rapid diffusion of vacancies from the surface into deeper layers and vice versa, in line with the low migration barriers obtained from DFT calculations (see Supporting Information, Figure S13), thereby sustaining continued surface reduction and promoting a more complete transformation, while also enabling accelerated surface reoxidation. Again, such behavior can be rationalized by comparing to the undoped system: There, the reduction-induced phase transition is accompanied by a 2.4% lattice expansion, reflecting the difference between CeO_2_(111) and a-Ce_2_O_3_(0001) lattice parameters.^31^ In contrast, the Sm-CeO_*x*_ islands already display a lattice parameter expanded to nearly that of hexagonal a-Ce_2_O_3_(0001) due to Sm incorporation, and no further change is detected upon transformation to the hexagonal phase. This pre-expanded lattice, arising from Ce^3+^ formation upon Sm incorporation, likely promotes the accelerated phase transition, as the doped islands have already accommodated the structural strain. In addition, ceria reduction proceeds through the formation and mobility of Ce^3+^-oxygen vacancy complexes,^15^ whose formation and migration energies are significantly lowered by Sm doping and related aliovalent substitutions.^70^

**Figure 8:**
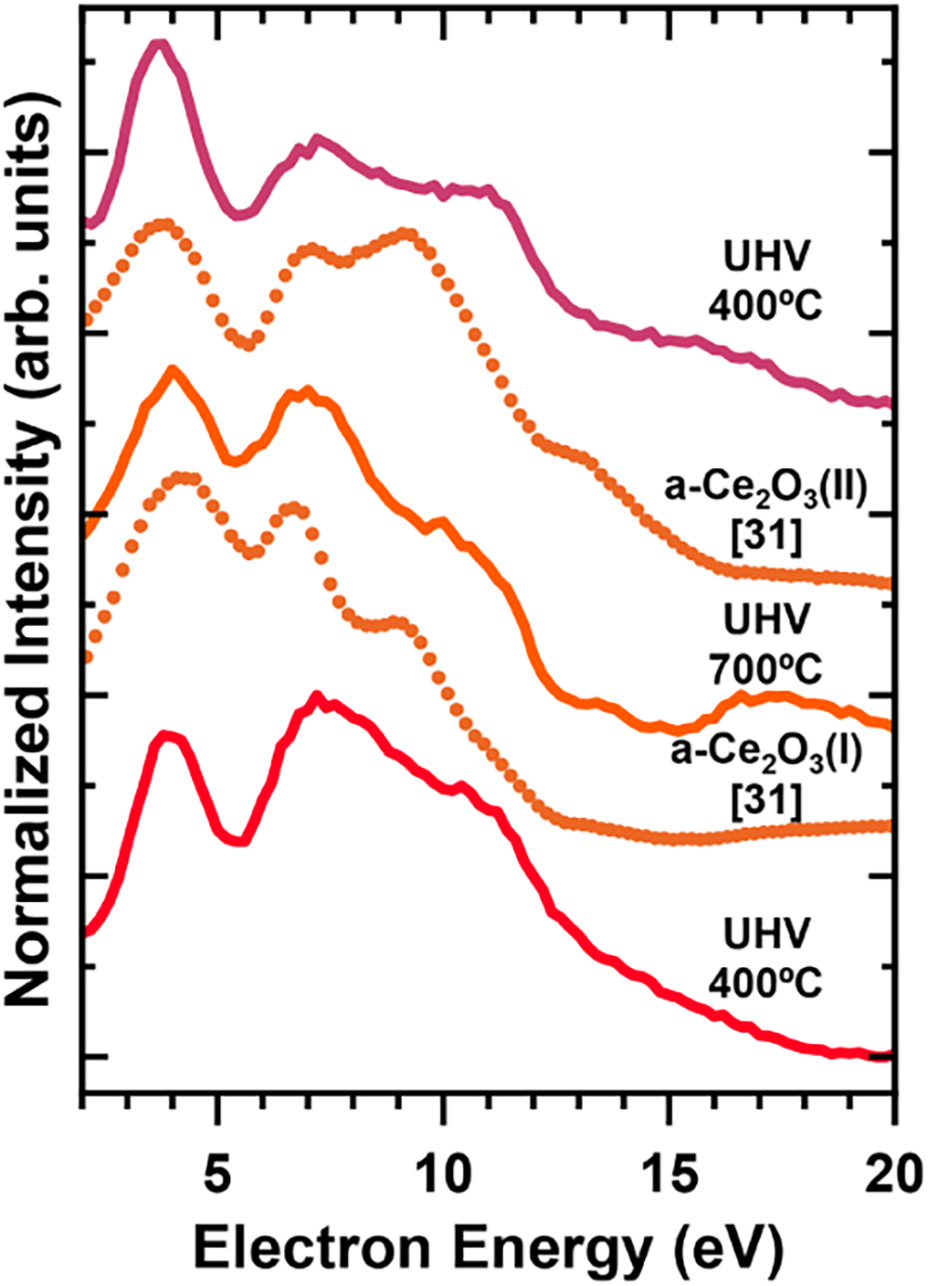
*I­(V)* curves measured on Sm-CeO*_x_* nanoislands before (red), during (orange), and after (magenta) annealing at 700 °C. For comparison, reference spectra for hexagonal reduced ceria are shown as orange dotted lines. Spectra are vertically shifted for clarity. Reference a-Ce_2_O_3_(I) and a-Ce_2_O_3_(II) adapted with permission from ref 31. Copyright [2017] [RSC Pub].

Summarizing the comparison between ceria with and without Sm-doping, our experimental findings and theoretical calculations highlight the dual role of Sm: it promotes extrinsic vacancy formation and enhanced vacancy mobility, while facilitating intrinsic vacancy formation through lattice expansion associated with Ce^3+^, enabling faster redox cycling than in undoped ceria, while simultaneously stabilizing the reduced lattice.

## Conclusions

By combining LEEM, μLEED, XAS-PEEM, XPEEM, and DFT, we resolve with nanometer-scale spatial resolution and electronic-state specificity the structural evolution and redox response of (111)-oriented CeO_2_ nanoislands doped with Sm on Ru(0001). Postannealing ceria with metallic Sm drives dopant incorporation (∼7 at%), simultaneously introducing extrinsic oxygen vacancies promoting Ce^3+^ formation that expands the fluorite CeO_2_(111) lattice toward that of hexagonal Ce_2_O_3_(0001). Under reducing conditions, this Ce^3+^-induced lattice expansion significantly lowers the formation energy of intrinsic oxygen vacancies, enabling their rapid generation and imparting markedly enhanced reducibility, as corroborated by DFT calculations.

Beyond modifying vacancy energetics, Sm doping also fundamentally reshapes the redox dynamics of ceria. Under molecular hydrogen, the Sm–CeO_*x*_ nanoislands rapidly develop ordered intrinsic vacancy superstructures and reach a partially reduced state on time scales dramatically shorter than those observed for undoped ceria. At elevated temperatures, the doped islands undergo a swift and complete transition to hexagonal Sm–Ce_2_O_3_ without the decomposition, Ce segregation, or metallic adlayer formation characteristic of the undoped system. These behaviors directly expose the stabilizing role of Sm, which promotes rapid vacancy formation while stabilizing the reduced lattice.

DFT analysis reveals that Sm doping influences oxygen-vacancy formation through two coupled effects. Aliovalent charge compensation stabilizes the first, extrinsic oxygen vacancy, whereas lattice expansion induced by Ce^3+^ formation is primarily responsible for lowering the formation energy of the additional intrinsic vacancy. The expanded lattice additionally accommodates the larger Ce^3+^ ions generated during reduction, mitigating local strain and sustaining continued vacancy formation under reducing conditions. This establishes a self-reinforcing mechanism in which Sm-induced vacancy formation leads to Ce^3+^ generation and lattice expansion, which in turn lowers the formation energy of additional intrinsic vacancies. Experimentally, these effects manifest as accelerated reduction kinetics and altered phase-transition pathways, consistent with a system in which vacancy creation and redistribution proceed more readily than in undoped ceria.

Taken together, these results establish Sm-CeO_*x*_/Ru­(0001) as a benchmark inverse-catalyst platform for disentangling how aliovalent dopants govern vacancy formation, oxygen mobility, and phase stability in ceria-based oxides. More broadly, they define a clear and general design principle for redox-active materials, showing that the concerted use of aliovalent doping and strain engineering enables control over vacancy energetics, ordering, and redox kinetics. This mechanistic framework provides a powerful foundation for engineering next-generation ceria-based catalysts and related reducible oxides for vacancy-driven transformations that require fast, robust, and reversible redox cycling.

## Materials and Methods

### Experimental Procedures

A Ru(0001) single crystal (Mateck) with a nominal orientation better than 0.1° was used as the substrate for all growth experiments. Initially, and between growth runs, the crystal was cleaned in situ by repeated cycles of annealing and flashing up to 1600 °C in an oxygen atmosphere of 1 × 10^–7^ mbar, until a clean, well-ordered, and flat surface was confirmed by low-energy electron microscopy (LEEM) and low-energy electron diffraction (LEED).

The growth experiments were performed using two commercial e-beam evaporators (Focus EFM 3) equipped with molybdenum crucibles loaded with metallic cerium (Alfa Aesar, 99.9%) and a metallic samarium rod (Alfa Aesar, 99.9%). For ceria growth, the Ru(0001) surface was exposed to 5 × 10^–7^ mbar O_2_ at 800 °C before and during deposition. After initial characterization at this temperature, the undoped CeO_2_ nanoislands were exposed to oxygen at 1 × 10^–7^ mbar to ensure full oxidation to CeO_2_. Then, metallic Sm was deposited onto CeO_2_/Ru­(0001) in UHV in the absence of oxygen.

Experiments were performed at the Nanospectroscopy beamline of the Elettra synchrotron in Trieste,^87^ equipped with a commercial spectroscopic photoemission LEEM (SPELEEM III, Elmitec GmbH). LEEM and μLEED measurements^60,88,89^ were performed using an internal electron source. X-ray absorption photoemission electron microscopy (XAS-PEEM) was carried out using synchrotron radiation, achieving a lateral resolution of approximately 30 nm.^90,91^

### Theoretical Models and Methods

Spin-polarized calculations were performed using the Vienna Ab initio Simulation Package (VASP, https://www.vasp.at, version 6.5.1)^92,93^ employing the frozen core projector-augmented wave (PAW) method^94^ and the generalized gradient approximation (GGA) of Perdew–Burke–Ernzerhof (PBE).^95^ The Ce (4f, 5s, 5p, 5d, 6s), Sm (4f, 5s, 5p, 5d, 6s) and O (2s, 2p) electrons were treated explicitly as valence states. For Sm, the pseudopotential assumed a Sm^3+^ configuration, with five of the six 4f electrons treated as core states. A Hubbard *U* correction of 4.5 eV was applied to the Ce 4f states,^96^ and the plane-wave cutoff was set to 500 eV for slab calculations.

The lattice parameter of bulk CeO_2_ (fluorite structure, Fm3̅m), lattice parameter was optimized using a 650 eV cutoff and a (5 × 5 × 5) Monkhorst–Pack *k*-point mesh,^97^ allowing full cell relaxation. The optimized lattice parameter was used in constructing four O–Ce–O trilayer-thick slab models of the stoichiometric CeO_2_(111) surface and reduced CeO_2–*x*_(111) surfaces with (2 × 2) periodicity. A vacuum space of at least 15 Å was included in all supercells to minimize spurious interactions between periodic images. Samarium doping was introduced by substituting one or two Ce atoms in the slab with Sm atoms. The Brillouin zone was sampled using a (3 × 3 × 1) *k*-point mesh.

During geometry optimization, all atoms in the bottom trilayer were fixed at their optimized bulk-truncated positions, while the remaining atoms were allowed to fully relax. The in-plane lattice parameters were kept fixed to those of stoichiometric bulk CeO_2_, thereby preventing changes in the cell vectors during relaxation. Total energies and forces were calculated with a precision of <1 × 10^–7^ eV and <|0.01| eV/Å for electronic and force convergence, respectively.^98^

Oxygen vacancy formation energies (*E*_*f*_) were computed for both undoped and Sm-doped surfaces as^80^

1 $$E_{f}=E_{\mathrm{red}}+\frac{1}{2}E_{O_{2}}-E_{\mathrm{ox}}$$

where *E*_red_ and *E*_ox_ are the total energies of the reduced and oxidized surfaces, respectively, and $E_{O_{2}}$ is the energy of an isolated molecule in the gas phase.

For clarity, oxygen vacancy configurations are denoted as follows:

- The subscript *S* or *SS* specifies the vacancy location: surface (S) or subsurface (SS).
- A pair of integers *n* and *m* in parentheses, e.g., $V_{\mathrm{SS}}^{\left(n,m\right)}$ , indicates the neighboring cationic coordination shells relative to the oxygen vacancy in which the Sm dopants are located. Here, 1 and 2 correspond to nearest–nearest-neighbor (NN) and next-nearest-neighbor (NNN) positions, respectively.
- A second pair of integers *n* and *m* in square brackets, e.g., $V_{\mathrm{SS}}^{[n,m]}$ , indicates the coordination shells relative to the vacancy in which the Ce^3+^, resulting from the formation of intrinsic oxygen vacancies, are located.
- Underlined integers denote that the corresponding Sm dopants and/or the reduced Ce^3+^ ions are located in the second trilayer of the ceria slab.

For example, $V_{\mathrm{SS}}^{(1,2)[2,2̲]}$ denotes a subsurface oxygen vacancy (SS) with one Sm^3+^ in a NN position and another in a NNN position relative to the vacancy, and with two Ce^3+^ in NNN sites, one in the first trilayer and the other one in the second trilayer (underlined).

Transition state (TS) structures were located using the climbing image nudged elastic band (CI-NEB) method^99^ under the DFT + U approximation, with 14 images for each reaction pathway. For all TS reported in this study, only one imaginary frequency was found.

## Supplementary Material







## Data Availability

The DFT data that support the findings of this study are available on Materials Cloud https://www.materialscloud.org/home with the identifier DOI: 10.24435/materialscloud:ya-z3. The data is also available from the authors upon reasonable request.

## Footnotes

1 Lattice parameter extracted using the CeO_2_(111) in-plane spacing as reference. Errors represent standard deviations from peak positions in all μLEED directions.

2 Because these measurements span several minutes, transient oxidation-state changes may further influence the *I­(V)* line shape; accordingly, this comparison is qualitative.

## Notes

Full LEEM time-accelerated movie recorded during CeO_2_ deposition at 800 °C in oxygen atmosphere on Ru(0001) (MP4)
Full LEEM time-accelerated movie recorded during Sm_2_O_3_ deposition at 800 °C in oxygen atmosphere on Ru(0001) (MP4)
Further content: spatially resolved XAS-PEEM images of Sm–CeO_x_ nanoislands; quantitative Sm concentration determination via XPEEM; structural characterization of Sm_2_O_3_/Ru­(0001); temperature effects on CeO_2_ nanoislands; comprehensive DFT + U calculations; a literature comparison of reported oxygen-vacancy formation energies in Ln-doped ceria; oxygen-vacancy formation energies for surface and subsurface vacancies in undoped and Sm-doped CeO_2_; effects of local Sm dopant configuration and surface cell size (2 × 2 vs 4 × 4); disentangling the roles of lattice expansion and dopant concentration on intrinsic vacancy formation; CI-NEB calculations of oxygen vacancy migration barriers and oxygen mobility (PDF)

## Abbreviations

LEEM: Low-energy electron microscopy
LEED: Low-energy electron diffraction
XAS-PEEM: X-ray absorption spectroscopy in photoemission electron microscopy
XPEEM: X-ray photoemission electron microscopy
DFT: Density functional theory
