# Supplementary material for: Samarium-Driven Vacancy Engineering in CeO2/Ru(0001) Inverse Catalysts
Source: Chem Mater. 2026 Jul 29;38(15):7590–601. doi: 10.1021/acs.chemmater.6c00161 (PMC13480377; doi:10.1021/acs.chemmater.6c00161)
Supplement: Supplementary file 1 [file cm6c00161_si_003.pdf]

Samarium-Driven Vacancy Engineering in CeO_2/Ru(0001) Inverse Catalysts – Supplementary Information

Raquel Sánchez-Barquilla[1], Alexander Contreras-Payares[2,3], Rudi Tschammer[1], Carlos Morales[1], Lars Buß[1], Björn Riedel[1], Iulia Cojocariu[4], Tevfik Onur Mentes[4], Matteo Jugovac[4], Pablo G. Lustemberg[2], Andrea Locatelli[4], M. Verónica Ganduglia-Pirovano*[2], and Jan Ingo Flege*[1]

[1]Applied Physics and Semiconductor Spectroscopy, Brandenburg University of Technology Cottbus-Senftenberg, 03046 Cottbus, Germany

[2]Instituto de Catálisis y Petroleoquímica, (ICP-CSIC), C/ Marie Curie 2, 28049 Madrid, Spain

[3]Ph.D. Theoretical Chemistry and Computational Modelling Doctoral School, Universidad Autónoma de Madrid, C/Francisco Tomás y Valiente 2, 28049 Ciudad Universitaria de Cantoblanco, Madrid, Spain

[4]Elettra-Sincrotrone Trieste, S.C.p.A, Strada Statale 14 - km 163.5 in AREA Science Park, Basovizza 34149, Trieste, Italy.

*Email: flege@b-tu.de, vgp@icp.csic.es

1. Caption and time series of supplementary movies

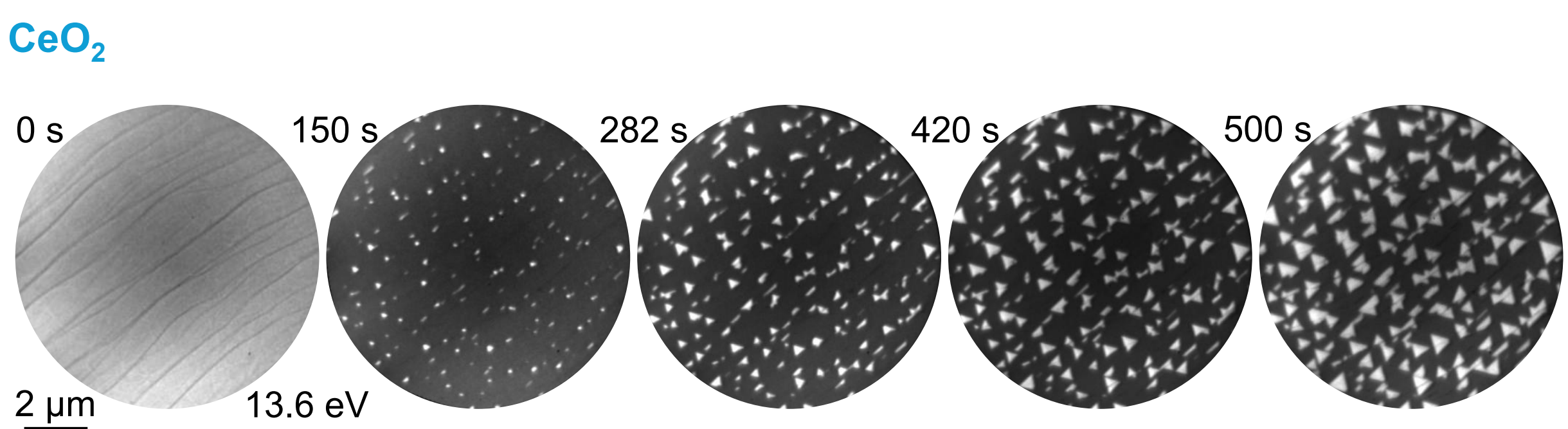

Figure S1: LEEM time series recorded during Ce deposition on Ru(0001) at 800 °C under an oxygen pressure $P_{O_2} = 5 \times 10^{-6}$ mbar (see movie S1 for the complete sequence). The elapsed time and electron energy are indicated in each frame. Field of view: 10 μm.

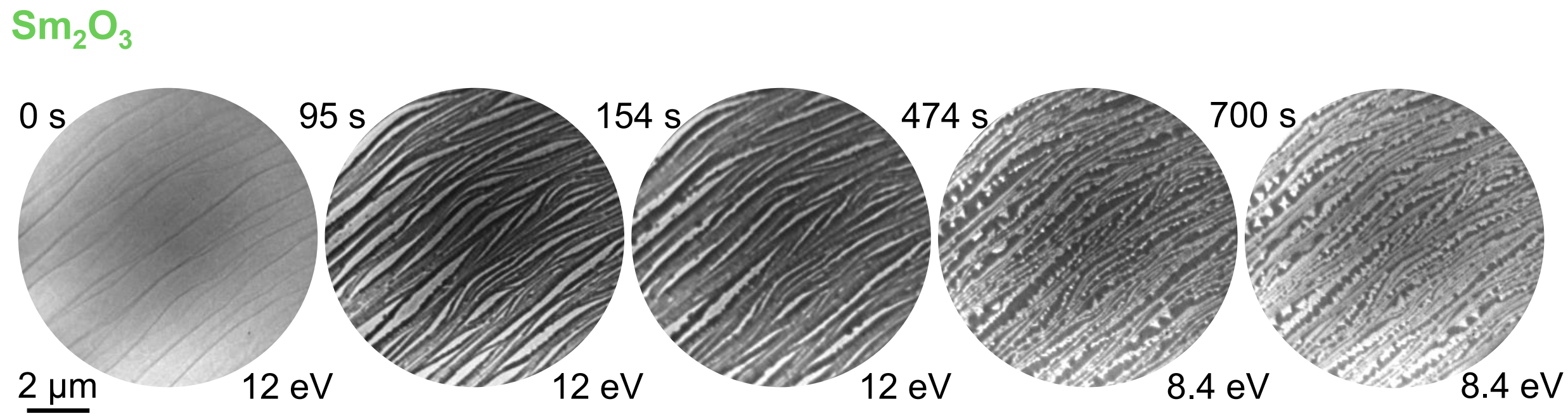

Figure S2: LEEM time series recorded during Sm deposition on Ru(0001) at 800 °C and oxygen pressure $P_{O_2} = 5 \times 10^{-6}$ mbar (see movie S2 for the complete sequence). The elapsed time and electron energy are indicated in each frame. Field of view: 10 μm.

2. XAS-PEEM images of Sm-CeO$_x$ islands

Fig. S3 shows spatially resolved XAS-PEEM images acquired at the Ce M_5 and Sm M_5 edges after post-annealing ceria islands with metallic samarium. The results reveal that Sm adsorption and incorporation occur predominantly on the ceria islands, leaving the Ru(0001) substrate uncovered. This confirms selective Sm incorporation into the ceria phase rather than deposition on the metal support. Representative spectra from these particles are presented in the main text (Fig. 4).

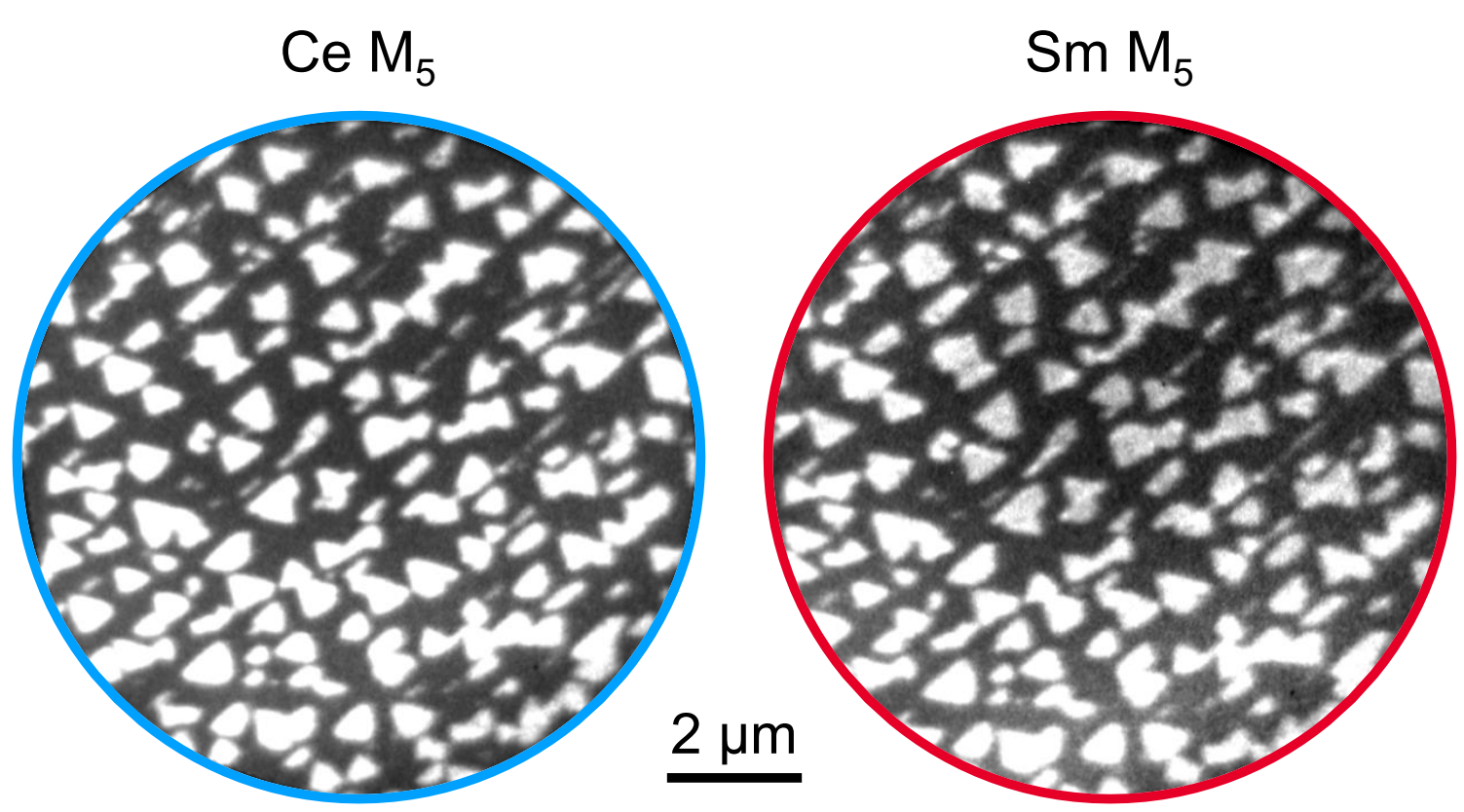

Figure S3: XAS-PEEM images acquired at the Ce M_5 edge (883 eV) and the Sm M_5 edge (1078 eV) for CeO_2 islands post-annealed with metallic samarium, showing the spatial distribution of Ce and Sm after incorporation.

3. Sm concentration in Sm-Ce nanoislands.

Figure S4 shows the XPS spectra measured in X-ray photoemission electron microscopy (XPEEM) mode at $h\nu = 195$ eV of the Sm 4d and Ce 4d region after background subtraction. The Sm content within the nanoislands was determined to be about 7% by comparing the intensities of the ceria (in blue) and samaria (in green), corrected by the cross section of both materials [1]. The fit model for the Ce 4d is constructed considering the relative energy shifts of the main peaks of Ce^{3+} and Ce^{4+} in Ref. [2]. The Sm 4d multiplet structure is more compact and allows for a rough fit model comprising two main components: a broad peak centered at approximately 132 eV, and a less intense shoulder shifted by approximately 5 eV. This calculation

also accounts for the asymmetry factors associated with the linear polarized light [3], and the direction of the emitted electrons.

This estimate of Sm concentration relative to cerium may be slightly underestimated ($< 5\%$) due to the low signal-to-noise ratio, complex background subtraction considering the transmission function of the instrument, partial overlap between the Ce 4d and Sm 4d components, and the lack of elaborated fitting models for these regions.

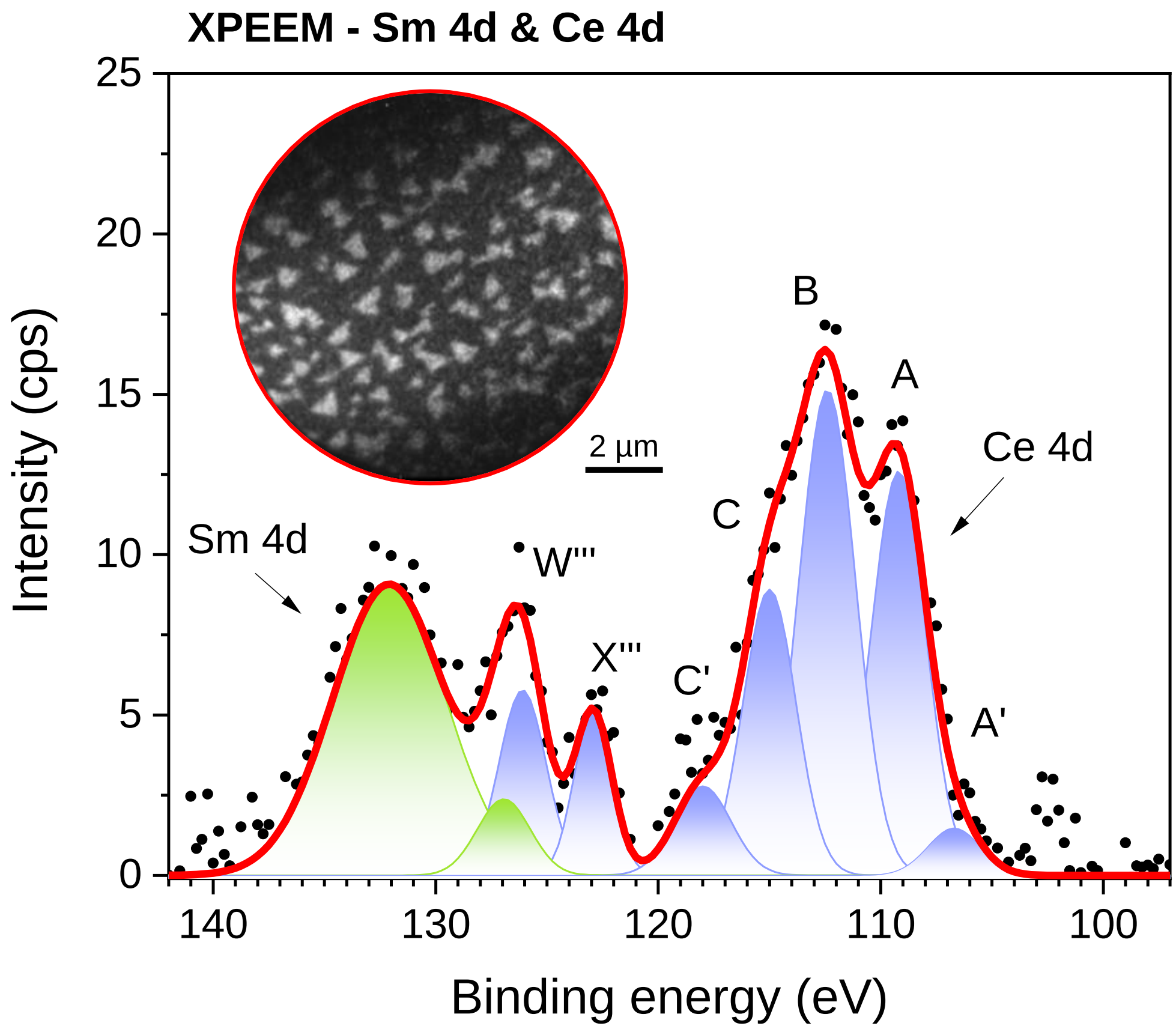

Figure S4: XPEEM measurement ($h\nu = 195$ eV) after Sm deposition, measured at 800 °C and under UHV conditions. Ce and Sm peaks are represented in blue and green, respectively. In the inset, XPEEM image at 110 eV binding energy.

4. Characterization of Sm_2O_3 on Ru(0001)

The inset of Fig. S5 shows a LEEM image recorded at 8.2 eV of Sm_2O_3 grown on Ru(0001). As in the case of CeO_2, described in the main text, the deposition was performed by evaporating metallic Sm in an oxygen atmosphere. First, the Ru(0001) substrate was heated to 800 °C and exposed to an oxygen atmosphere at the same pressure as used for ceria (5×10^{-7} mbar) to ensure complete oxidation of the substrate and to enable direct comparison with the Sm deposited on CeO_2 at the same temperature. The μLEED pattern is shown in the left inset. From this pattern, a surface lattice parameter of 3.70 ± 0.10 Å is obtained.

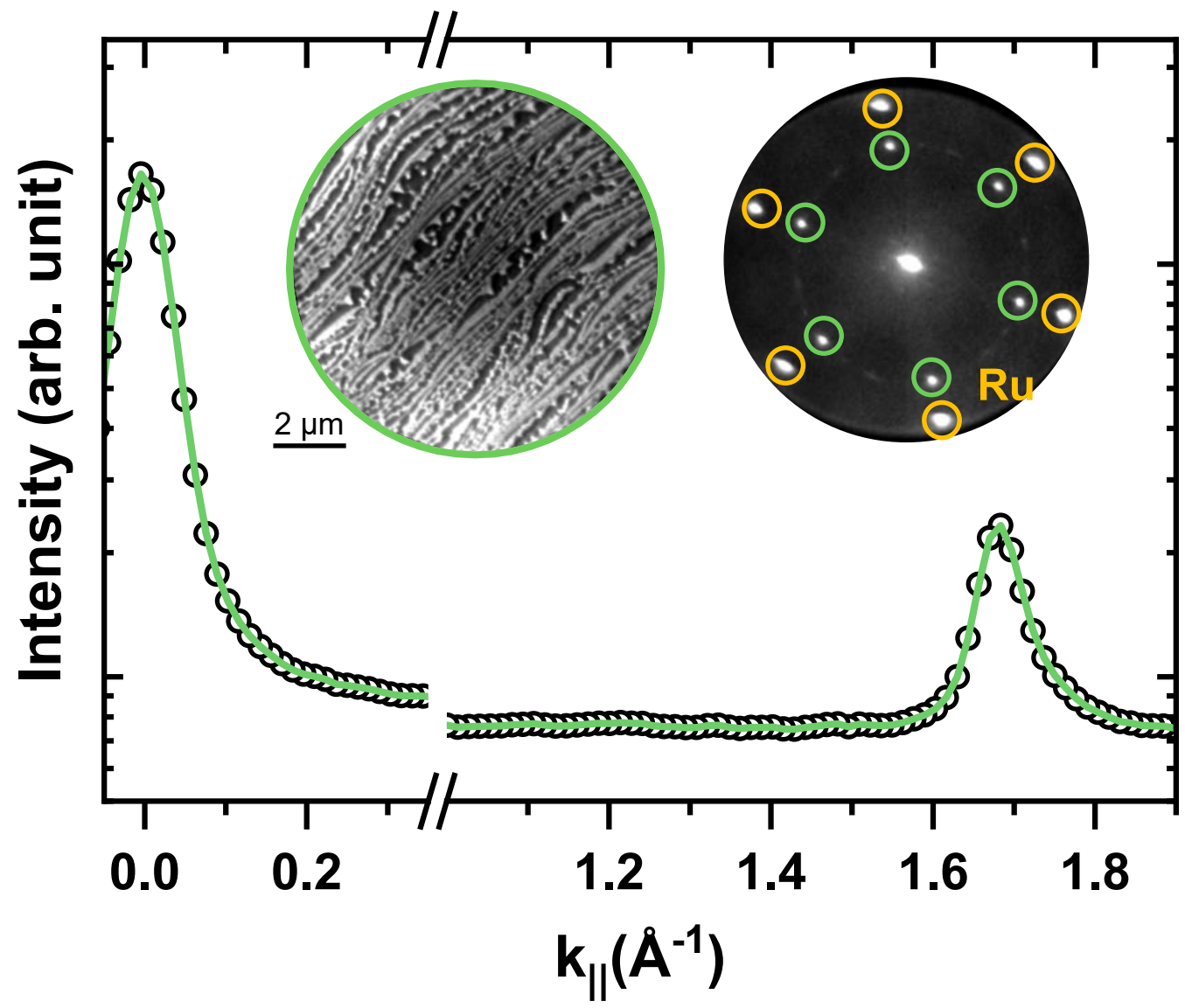

Figure S5: μLEED intensity profile extracted along one reciprocal-space direction of the LEED pattern shown in the right inset. The left inset shows a LEEM image acquired at 8.2 eV.

5. Temperature effect of CeO_2 nanoislands.

Comparison between the *I(V)* curves of the CeO_2 nanoislands measured immediately after CeO_2 growth (i.e., at $5 \cdot 10^{-7}$ mbar of oxygen pressure and 800 °C), and in UHV conditions at the same temperature. This control measurement shows that removing the oxygen pressure in the chamber does not lead to measurable reduction of the CeO_2 nanoislands. Thus, the change in the *I(V)* curve discussed in Fig. 3 is due to the Sm deposition.

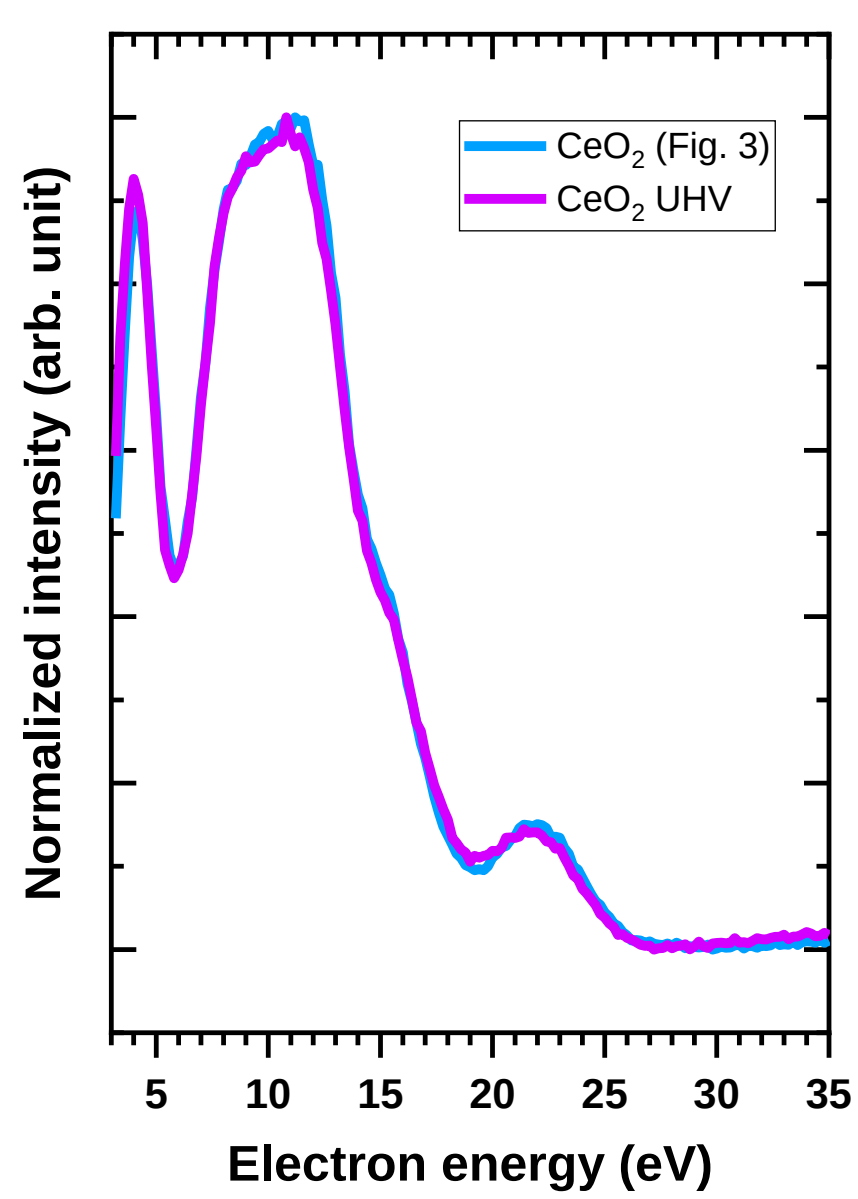

Figure S6: Comparison between the IV-LEEM immediately after CeO_2 growth ($5 \cdot 10^{-7}$ mbar and 800 °C) and in UHV conditions at the same temperature.

6. Density-functional theory calculations

Table S1: Summary of reported DFT+U oxygen-vacancy formation energies (E_f) in Ln-doped CeO_2 (Ln = Sm, Gd, La), with emphasis on Sm-doped systems. Extrinsic vacancies are dopant-associated; intrinsic vacancies are additional vacancies beyond dopant compensation. NN and NNN denote nearest- and next-nearest-neighbor cation positions relative to the oxygen vacancy (V_O).

System	No. of Ln	Vacancy type	Ln / Ce^{3+} position relative to V_O	E_f (eV)	Compensation[a]
La-doped	1	Surface (extrinsic)	La: NN; Ce^{3+}: not reported	1.36[b]	Partial
	2	Surface (extrinsic)	2La: NNN; Ce^{3+}: NN	0.90[c]	Partial
	1	Surface (extrinsic)	La: NNN; Ce^{3+}: NNN	0.49[d]	Partial
	2	Surface (extrinsic)	2La: NNN	-0.94[d]	Full
Ga-doped	2	Subsurface (extrinsic)	2Ga: NN	-1.11[e]	Full
	4	2 Surface (extrinsic)	2Ga: NN per V_O	-1.85[e]	Full
Sm-doped	1	Bulk (extrinsic)	Independent of Sm position; Ce^{3+}: not reported	≈ -0.30[f]	Partial
	2	Bulk (extrinsic)	2Sm: NN	≈ -0.80[g]	Full
	2	Bulk (extrinsic)	2Sm: NN	-0.70[h]	Full
	2	Bulk (intrinsic)	2Sm: NNN; Ce^{3+}: NN	2.76[i]	Full
	2	Bulk (extrinsic)	2Sm: NN	-0.90[j]	Full
	2	Bulk (intrinsic)	2Sm: NN; Ce^{3+}: not reported	2.85[j]	Full
	1	Surface (extrinsic)	Sm: NN; Ce^{3+}: not reported	1.68[k]	Partial

[a] **Charge compensation:** *Full* indicates that the vacancy charge is fully compensated by dopants (extrinsic vacancies) or by Ce^{3+} ions (intrinsic vacancies). *Partial* indicates incomplete dopant compensation, requiring the formation of one Ce^{3+} ion. Entries marked as "Ce^{3+}: not reported" indicate that the referenced study did not explicitly resolve or discuss the localization of the reduced Ce^{3+} species.
[b] Hu *et al.*, J. Phys. Chem. C **115** (2011) 3065–3074.
[c] Yeriskin *et al.*, J. Phys.: Condens. Matter **22** (2010) 135004.
[d] Gao *et al.*, Appl. Surf. Sci. **497** (2019) 143732.
[e] Aparicio-Angles *et al.*, Chem. Mater. **27** (2015) 7910–7917.
[f] Zhang *et al.*, Inorg. Chem. **57** (2018) 12690–12696.
[g] Alaydrus *et al.*, Phys. Chem. Chem. Phys. **18** (2016) 12938.
[h] Andersson *et al.*, Proc. Natl. Acad. Sci. USA **103** (2006) 3518–3521.
[i] Lucid *et al.*, J. Phys. Chem. C **120** (2016) 23430–23440.
[j] An *et al.*, Appl. Surf. Sci. **676** (2024) 161028.
[k] Liu *et al.*, Appl. Surf. Sci. **576** (2022) 151803.

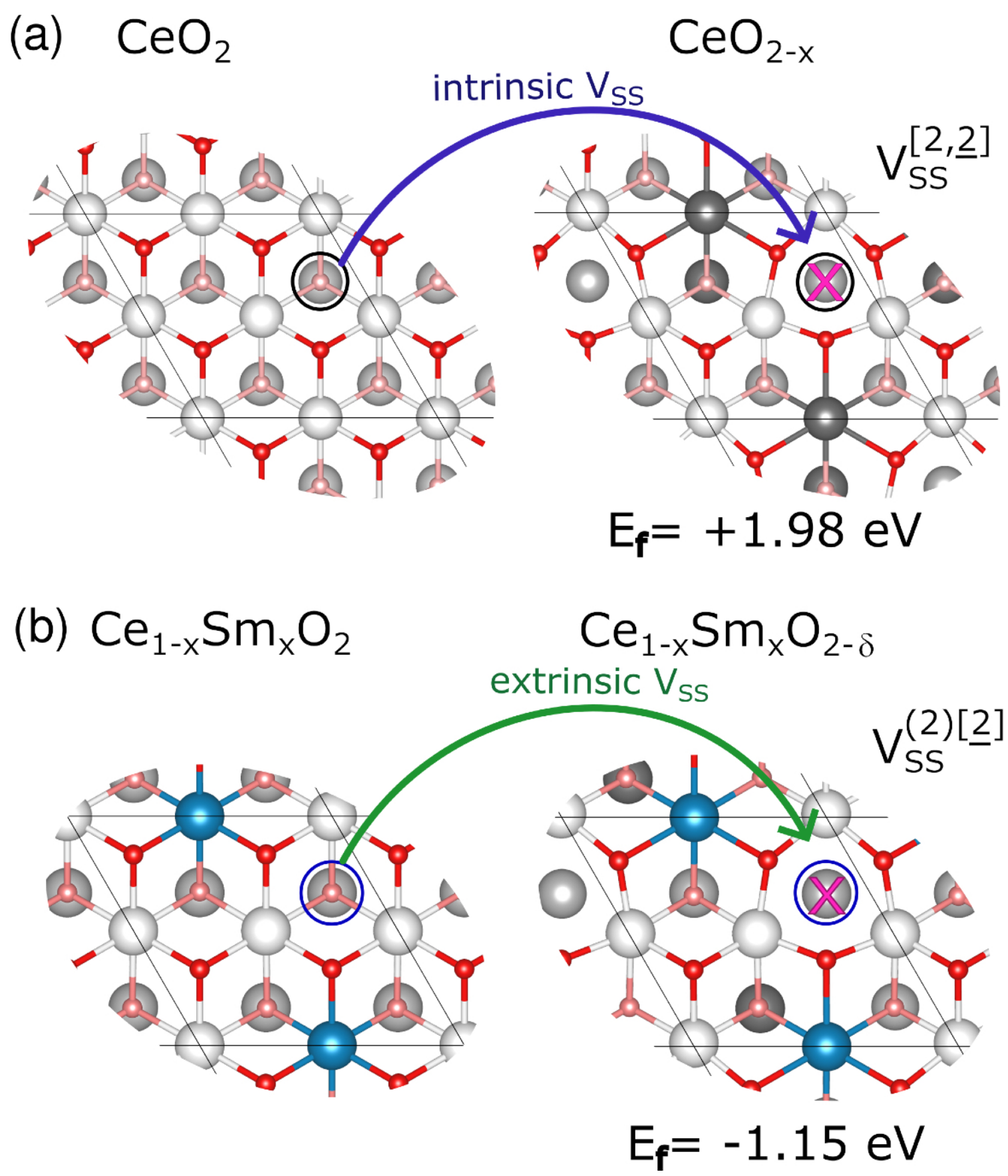

Figure S7: Subsurface oxygen vacancy formation in $CeO_2(111)$ surfaces (a) without and (b) with Sm doping, obtained from DFT calculations. In both cases, the vacancy corresponds to the most stable configuration, with excess electrons localized at next-nearest-neighbor (NNN) Ce cations. In panel (b), substitution of one Ce atom by Sm^{3+} cation partially compensates the excess charge, thereby lowering the vacancy formation energy and stabilizing Ce^{3+} formation. **Color code:** Ce^{4+} in white (light gray for Ce^{4+} in lower CeO_2 trilayers), Ce^{3+} (dark gray), Sm^{3+} (blue), O (surface red, subsurface:light red), vacancy site (crossed circle). For clarity, only atoms in the first O–Ce–O trilayer (TL) and cations in the second TL are shown.

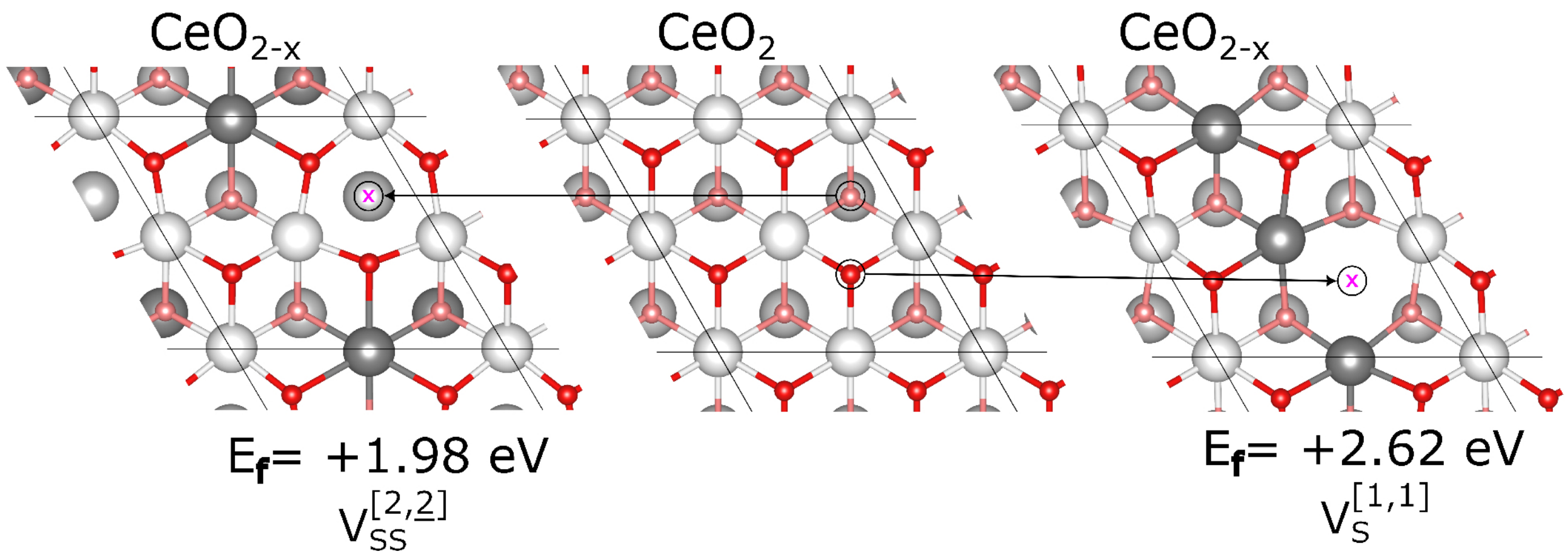

Figure S8: DFT-calculated formation energies for surface and subsurface oxygen vacancies in undoped $CeO_2(111)$. The subsurface vacancy $V_{SS}^{[2,\underline{2}]}$ is the most stable configuration, with excess electrons localized at next-nearest-neighbor Ce cations. Higher-energy vacancy configurations are included for comparison. **Color code:** Ce^{4+} in white (light gray for Ce^{4+} in lower CeO_2 trilayers), Ce^{3+} (dark gray), Sm^{3+} (blue), O (surface red, subsurface:light red), vacancy site (crossed circle). For clarity, only atoms in the first O–Ce–O trilayer (TL) and cations in the second TL are shown.

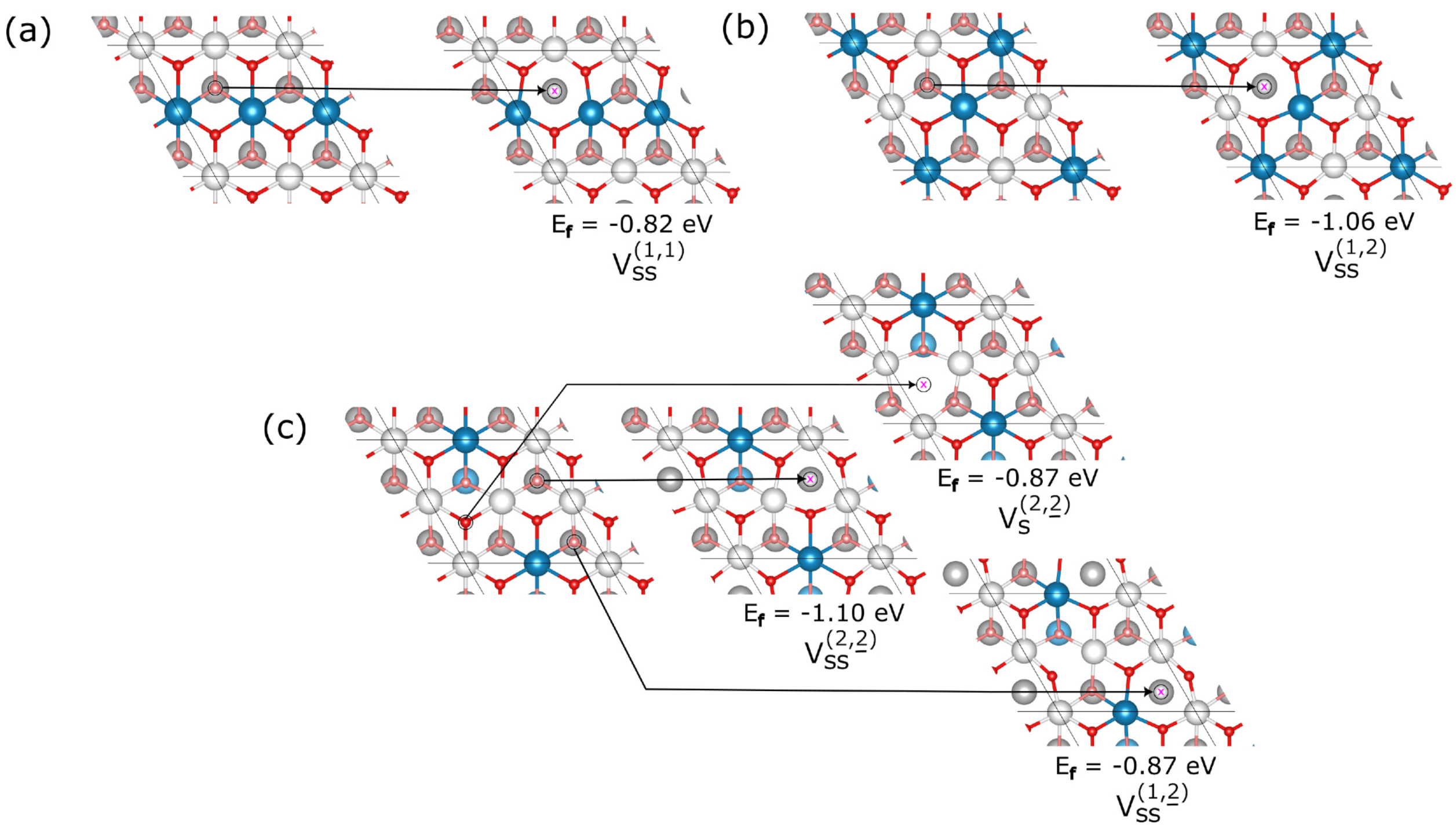

Figure S9: DFT-optimized structures of $CeO_2(111)$ doped with two Sm^{3+} cations, illustrating three distinct configurations of an extrinsic oxygen vacancy. The computed formation energy of each configuration is reported relative to the fully oxidized Sm-doped surface. **Color code:** Ce^{4+} in white (light gray for Ce^{4+} in lower CeO_2 trilayers), Ce^{3+} (dark gray), Sm^{3+} (blue), O (surface red, subsurface:light red), vacancy site (crossed circle). For clarity, only atoms in the first O–Ce–O trilayer (TL) and cations in the second TL are shown.

Figure S9 examines the effect of the local dopant environment on extrinsic oxygen vacancy formation in $CeO_2(111)$ doped with two Sm^{3+} cations. In all configurations, the vacancy is fully charge-compensated by the two Sm^{3+} dopants, and no Ce^{3+} species are formed. In configurations (a) and (b), both Sm^{3+} ions are located in the first ceria trilayer and the vacancy lies in the subsurface. In configuration (a), the vacancy is coordinated by two Sm^{3+} and two Ce^{4+} nearest neighbors (NN), denoted $V_{SS}^{(1,1)}$. In configuration (b), it is coordinated by one Sm^{3+} and three Ce^{4+} NN, with the second Sm^{3+} occupying a next-nearest-neighbor (NNN) position, $V_{SS}^{(1,2)}$. In configurations (c), one Sm^{3+} is located in the first ceria trilayer and the other one in the second. Among these arrangements, two correspond to subsurface vacancies and one to a surface vacancy. For the subsurface cases, one configuration has the vacancy coordinated exclusively by four Ce^{4+} cations, with both Sm^{3+} occupying NNN positions, $V_{SS}^{(2,\underline{2})}$, while the other is coordinated by one Sm^{3+} and three Ce^{4+} NN, with the Sm^{3+} in the second trilayer occupying a NNN site, $V_{SS}^{(1,\underline{2})}$. In the surface vacancy case, the vacancy is coordinated exclusively by three Ce^{4+} cations, with both Sm^{3+} in NNN positions, $V_{S}^{(2,\underline{2})}$

These results demonstrate that the spatial distribution of Sm^{3+} relative to the vacancy strongly influences its stability. Moreover, consistent with the behavior of Ce^{3+} in undoped ceria, the most favorable Sm^{3+} positions are at NNN sites. Notably, the subsurface vacancy $V_{SS}^{(2,\underline{2})}$, is 0.4 eV more stable than the corresponding surface vacancy, $V_{S}^{(2,\underline{2})}$, highlighting the thermodynamic preference for vacancy formation in the subsurface layer when full compensation is achieved by NNN Sm dopants.

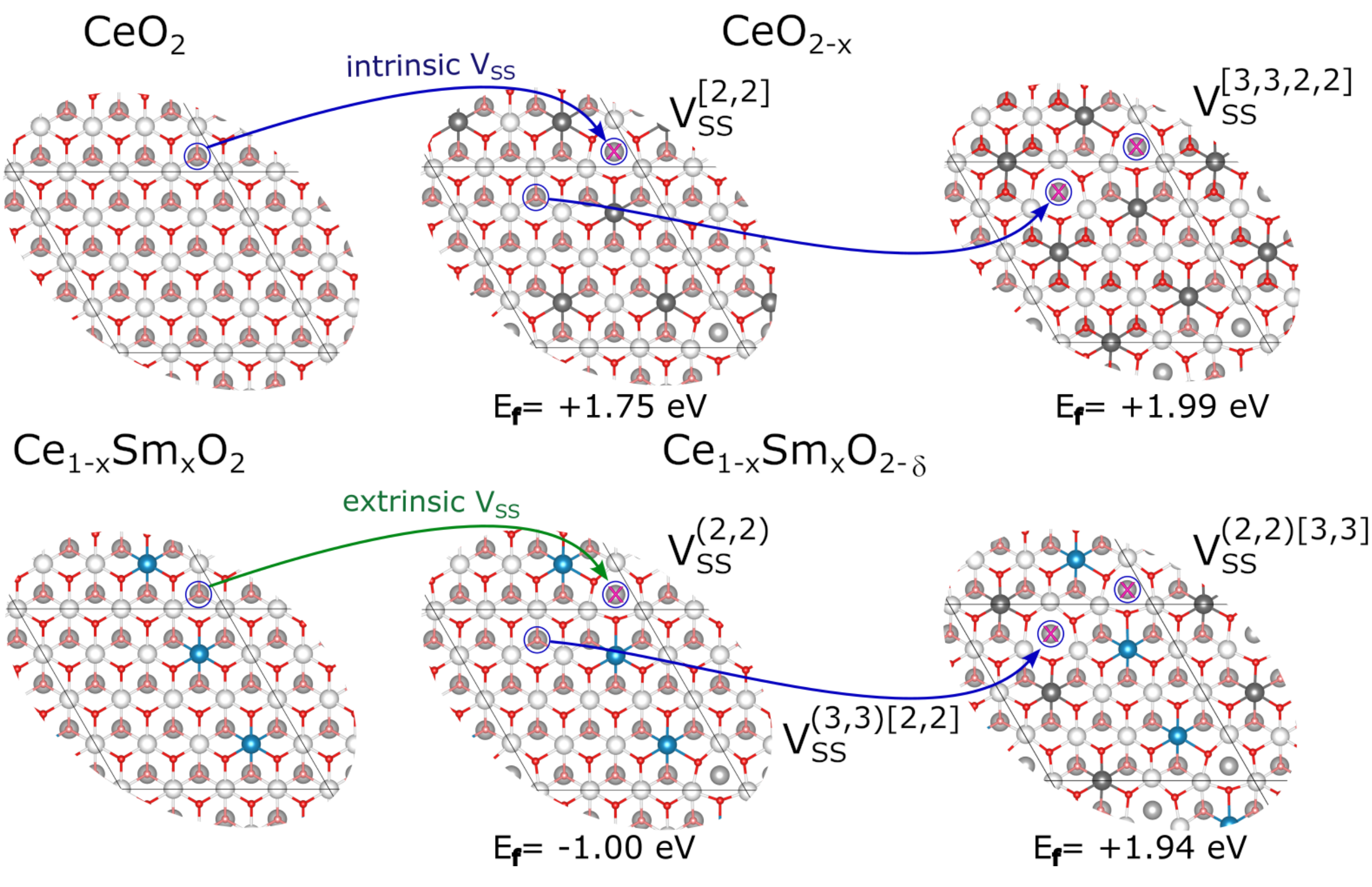

Figure S10: Effect of a larger surface unit cell (4×4) on oxygen vacancy formation in undoped and Sm-doped $CeO_2(111)$ surfaces.

To investigate the effect of reduced dopant and vacancy surface coverage, the surface model was expanded from a (2×2) to a (4×4) periodicity, and oxygen vacancy formation was compared for both undoped and Sm-doped $CeO_2(111)$. All calculations were performed using a $1 \times 1 \times 1$ **k**-point mesh.

In the Sm-doped system, the formation of an extrinsic subsurface oxygen vacancy with both Sm^{3+} dopants occupying NNN sites ($V_{SS}^{(2,2)}$) remains energetically favorable, with a formation energy of -1.00 eV. When a second vacancy is created far from the dopants ($V_{SS}^{(3,3)[2,2]}$), the formation energy is slightly lower than that

of the equivalent intrinsic vacancy in the undoped CeO_2 surface ($V_{SS}^{[2,2,3,3]}$). These results are consistent with those obtained using the (2×2) surface model (see main text and Figure S9 and S11), indicating that the qualitative trends in vacancy energetics are robust with respect to surface unit cell size.

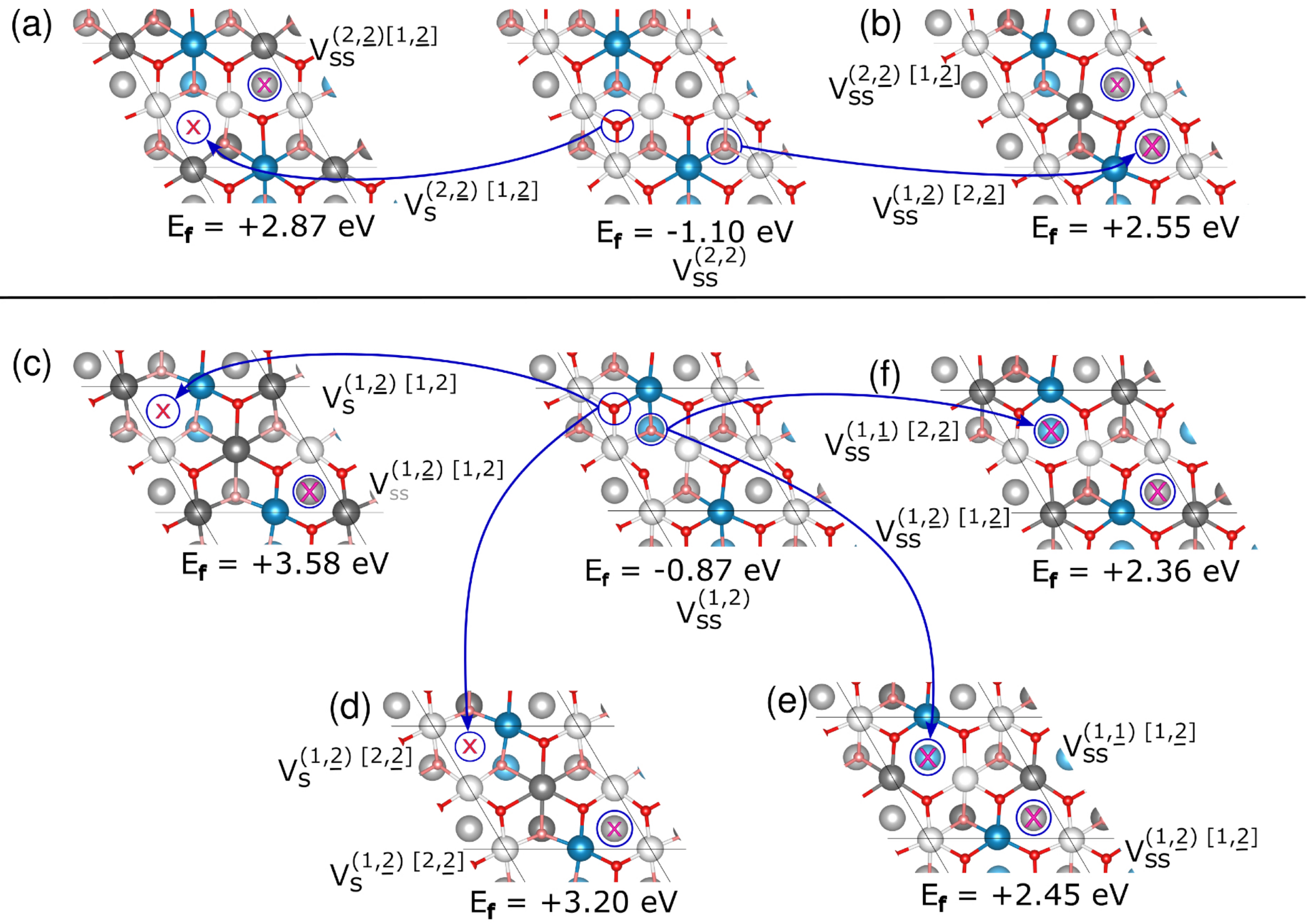

Figure S11: DFT-optimized configurations of Sm-doped $CeO_2(111)$. (a, b) Configurations starting from $V_{SS}^{(2,\underline{2})}$, with the second vacancy placed at the surface (a) or subsurface (b). (c–f) Configurations starting from $V_{SS}^{(1,\underline{2})}$, exploring different possible sites for the second vacancy. The corresponding formation energies are indicated, showing that subsurface intrinsic vacancies (particularly panel f) are thermodynamically preferred. **Color code:** Ce^{4+} (light gray), Ce^{3+} (dark gray), Sm^{3+} (blue), O (surface red, subsurface:light red), vacancy site (crossed circle). For clarity, only atoms in the first O–Ce–O trilayer (TL) and cations in the second TL are shown.

Figure S11 examines the relative stability of intrinsic oxygen vacancies in Sm-doped $CeO_2(111)$, where two Sm^{3+} dopants are compensated by one extrinsic vacancy. Two representative arrangements of the Sm dopants are considered: both in next-nearest-neighbor (NNN) positions relative to the vacancy ($V\mathrm{SS}^{(2,2)}$, top row, panels a–b), or one in a nearest-neighbor (NN) and one in a NNN position ($V_{SS}^{(1,2)}$, bottom row, panels c–f).

In both cases, one Sm^{3+} cation occupies a NNN site relative to the vacancy, while the second Sm^{3+} is either also in a NNN position (top row) or in a NN position (bottom row). This difference in dopant placement has a pronounced effect on the stability of the subsequently formed intrinsic vacancy.

In the top row, both Sm^{3+} cations are located at NNN positions relative to the initial subsurface vacancy. Two possible second vacancies are considered: a surface vacancy ($V_S^{(2,\underline{2})[1,\underline{2}]}$, panel a) and a subsurface vacancy ($V_{SS}^{(1,\underline{2})[2,\underline{2}]}$, panel b). The subsurface configuration is more stable by 0.32 eV..

For $V_{SS}^{(2,2)}$ (panels a–b), the additional intrinsic vacancy can thus form either the surface or the subsurface, with a clear energetic preference for the subsurface site.

For $V_{\mathrm{SS}}^{(1,2)}$ (panels c–f), multiple placements of the intrinsic vacancy were tested. The most stable configuration is $V_{\mathrm{SS}}^{(1,1)[2,2]}$ (panel f), with a formation energy of 2.36 eV, where both Sm^{3+} occupy NN positions while the Ce^{3+} ions localize at preferred NNN sites. Comparisons with panels c and d, which share similar Sm geometries but less favorable Ce^{3+} localization, emphasize that Ce^{3+} positioning plays a dominant role over Sm proximity in stabilizing intrinsic vacancy configurations.

Overall, these results confirm that subsurface intrinsic vacancies are thermodynamically preferred, and that the most stable double-vacancy configuration (panel f) arises from the less stable initial extrinsic geometry $V_{\mathrm{SS}}^{(1,2)}$.

Effect of lattice expansion and dopant concentration on intrinsic vacancy formation

To further clarify the respective roles of electronic effects and lattice expansion, we evaluated intrinsic oxygen vacancy formation under different structural and compositional conditions. We considered the formation of an intrinsic surface oxygen vacancy in a reduced slab ($Ce_{22}^{3+}Ce_{2}^{4+}Sm_{24}^{3+}O_{73} \rightarrow Ce_{24}^{3+}Sm_{24}^{3+}O_{72}$) both at the lattice parameter of stoichiometric CeO_2 and under lattice expansion of 3.4%, corresponding to the calculated lattice parameter of bixbyite Ce_2O_3. In line with the previous results, a substantial decrease in vacancy formation energy is observed upon lattice expansion. Moreover, maintaining the same lattice expansion (3.4%) while reducing the Sm concentration yields a similar vacancy formation energy, confirming that the effect is primarily governed by lattice expansion rather than by the specific dopant concentration (Figure S12).

Taken together, these results show that the reduction in vacancy formation energy is mainly driven by lattice expansion. However, since this expansion originates from the electronic changes associated with Sm^{3+} incorporation, namely the formation of Ce^{3+}, the two effects are inherently coupled. The enhanced reducibility of intrinsic vacancies is therefore primarily governed by lattice expansion, which itself originates from the electronic changes induced by Ce^{3+} doping.

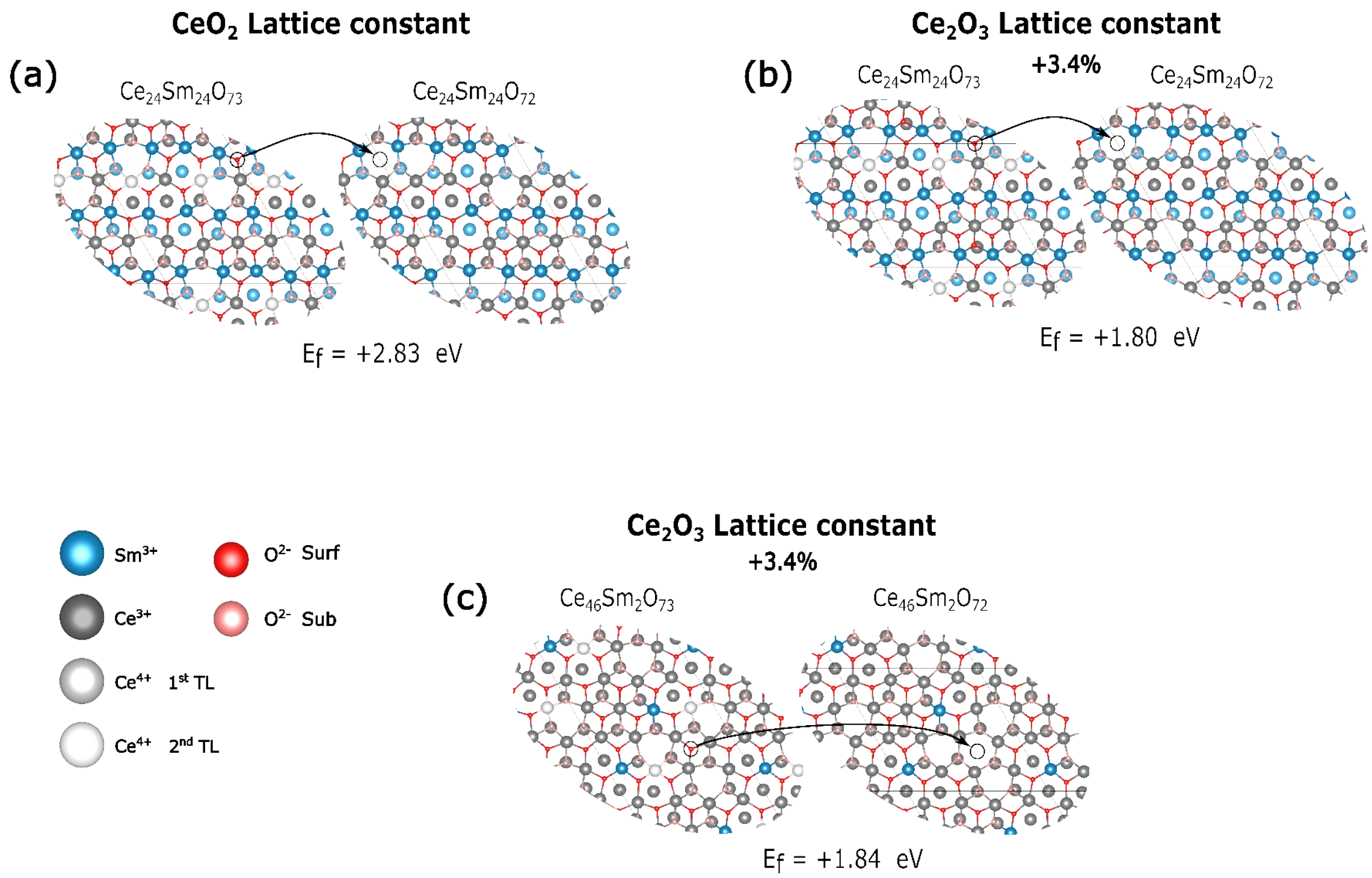

Figure S12: Intrinsic surface oxygen vacancy formation energy in Sm-doped CeO_2(111) as a function of lattice expansion and dopant concentration. (a) Vacancy formation at the lattice parameter of stoichiometric CeO_2. (b) Vacancy formation under lattice expansion of 3.4% representative of reduced ceria. (c) Same lattice expansion with reduced Sm concentration. The results show that lattice expansion leads to a substantial decrease in vacancy formation energy, whereas variations in dopant concentration at fixed expansion have a comparatively minor effect on the vacancy formation energy. For clarity, only atoms in the first O–Ce–O trilayer (TL) and cations in the second TL are shown.

Oxygen vacancy migration in Sm-doped CeO_2(111)

To support the interpretation of vacancy mobility discussed in the main text, we explicitly evaluated oxygen vacancy migration barriers using density functional theory (DFT) calculations and the climbing-image nudged elastic band (CI-NEB) method with 14 intermediate images.

We considered oxygen diffusion between subsurface and surface sites in Sm-doped CeO_2(111), which is equivalent to surface-to-subsurface vacancy migration, i.e., vacancy motion occurs in the direction opposite to oxygen diffusion. Calculations were performed both at the lattice parameter of stoichiometric CeO_2 and under lattice expansion representative of the doped system.

The calculated minimum-energy pathways are shown in Figure S13. In both cases, the diffusion proceeds via a single transition state (TS), confirmed by the presence of one imaginary vibrational frequency. The activation barrier for oxygen diffusion from subsurface to surface sites is low, amounting to 0.06 eV at the lattice parameter of CeO_2 and 0.11 eV under lattice expansion (3.8%). Lattice expansion significantly modifies the relative stability of the initial and final states along the diffusion pathway, thereby reducing the reverse barrier (surface-to-subsurface diffusion), from approximately 0.9 eV in the unexpanded lattice to $\sim$0.4 eV upon expansion. As a result, vacancy exchange between surface and subsurface sites is facilitated in both directions.

These results indicate that vacancy migration barriers are intrinsically low in both cases, consistent with previous theoretical studies on reduced ceria systems, and enabling rapid vacancy redistribution between surface and subsurface regions under the experimental conditions.

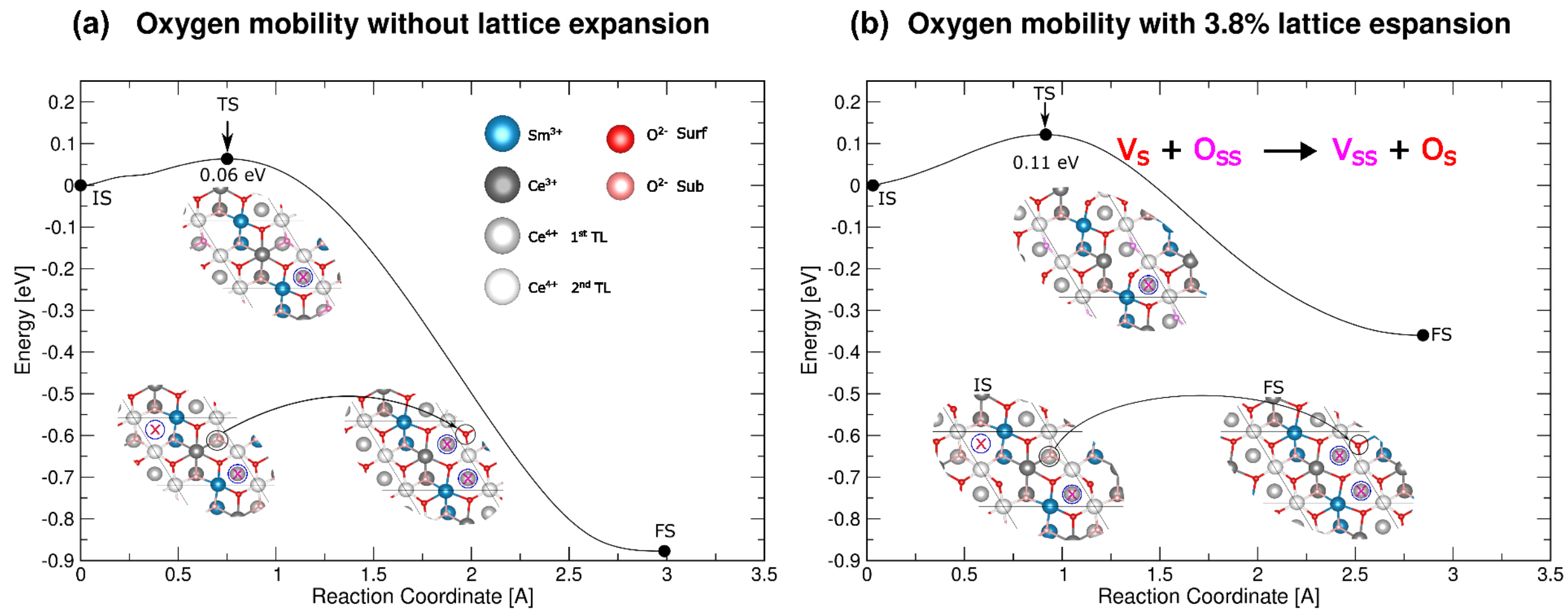

Figure S13: Minimum-energy pathways for oxygen diffusion between subsurface (initial state, IS) and surface sites (final state, FS) in Sm-doped $CeO_2(111)$. (a) At the lattice parameter of stoichiometric CeO_2 and (b) with a 3.8% lattice expansion representative of the doped system. The corresponding activation barriers are 0.06 eV and 0.11 eV, respectively. Transition states (TS) are indicated. For clarity, only atoms in the first O–Ce–O trilayer (TL) and cations in the second TL are shown. **Color code:** Ce^{4+} (light gray), Ce^{3+} (dark gray), Sm^{3+} (blue), O (surface red, subsurface:light red), vacancy site (crossed circle). For clarity, only atoms in the first O–Ce–O trilayer (TL) and cations in the second TL are shown.

References

(1) Yeh, J.; Lindau, I. *Atomic Data and Nuclear Data Tables* **1985**, *32*, 1–155.

(2) Mullins, D.; Overbury, S.; Huntley, D. *Surface Science* **1998**, *409*, 307–319.

(3) Cooper, J. W. *Physical Review A* **1993**, *47*, Publisher: American Physical Society, 1841–1851.
